# Effects and moderators of a 16-week intervention on contextual physical activity in adults with moderate to severe depressive symptoms: short and long-term results of a randomized clinical trial

**DOI:** 10.1186/s12966-026-01950-9

**Published:** 2026-07-21

**Authors:** Cecília Bertuol, Kelly Samara Silva, Marcus Vinicius Veber Lopes, Deborah Kazimoto Alves, Caroline Soares da Silva, Marcelo Baggio do Amaral, Anne Ribeiro Streb, Giovani Firpo Del Duca

**Affiliations:** 1 Physical Education Department, Federal University of Santa Catarina, Florianópolis, 88040-900 Brazil; 2 CHEO Research Institute, Ottawa, ON K1H 8L1 Canada; 3 Postgraduate Program in Public Health, State University of Ceará, Fortaleza, 60714-903 Brasil

**Keywords:** Active behavior, Adults, Clinical trial, Depressive symptoms

## Abstract

**Background:**

Depression is associated with reduced physical activity (PA), and evidence on the long-term effects of interventions and their potential moderators remains limited. Thus, this study aimed to evaluate the short- (post-intervention) and long-term (six-month follow-up) effects of a PA-promoting intervention on contextual PA and to explore potential moderators in adults with depressive symptoms.

**Methods:**

A randomized, controlled, and assessor-blinded clinical trial was conducted with adults presenting depressive symptoms. The intervention lasted 16 weeks, with two sessions per week. Strategies were grounded in Self-Determination Theory and aimed to increase participants’ awareness of their lifestyle, primarily focusing on PA and its relationship with depression. Activities included lectures, group discussions, interactive activities with family and friends, and PA practices in different contexts. Intensity-specific PA was assessed using wrist-worn accelerometers, and contextual PA (by type and domain) was self-reported. Generalized estimating equations were used to assess intervention effects. Potential moderators (sex and age) were examined in exploratory analyses using three-way interaction terms.

**Results:**

A total of 78 individuals (76.9% women, mean age 37.5 ± 10.2) were equally randomized into control (CG) and intervention (IG) groups. No significant group-by-time interactions were identified in the intention-to-treat analyses. However, per-protocol analysis suggested that the intervention increased leisure-time PA compared to controls (*p* = 0.004), with short-term increases (between-group changes of 119.6 min/week) and maintenance at follow-up (83.6 min/week). Exploratory moderator analyses indicated potential differences in intervention effects according to sex and age.

**Conclusion:**

Although no significant effects were identified in the intention-to-treat analyses, per-protocol findings suggested potential increases in leisure-time PA among participants with higher adherence. Exploratory findings indicate possible variation according to sex and age, but these results should be interpreted with caution and considered hypothesis-generating. Overall, the findings highlight the importance of strategies to improve adherence in PA interventions targeting adults with depressive symptoms.

**Trial registration:**

Brazilian Registry of Clinical Trials (ReBEC) RBR7466htj, April, 17 2023. Retrospectively registered.

**Supplementary Information:**

The online version contains supplementary material available at https://doi.org/10.1186/s12966-026-01950-9.

## Background

Evidence on the benefits of physical activity (PA) for individuals with chronic noncommunicable diseases has grown substantially in last decade [1–3]. Among adults with depression or depressive symptoms, PA has gained prominence not only for its preventive effects but also as a treatment strategy [4–8]. Despite this, its regular practice as an adjunctive resource for mental health remains under recognized in many clinical guidelines [9, 10]. This lack of recognition is compounded by the heterogeneity of interventions and the varied contexts in which they are implemented [11], making it challenging to identify the most effective strategies for promoting PA that yield a meaningful and sustained impact in adults with this clinical condition.

Systematic reviews with meta-analyses support the hypothesis that PA is associated with a reduced risk of developing depression [12, 13]. Conversely, the reverse relationship may also occur, with individuals experiencing depressive symptoms being less likely to engage in PA or to meet recommended levels [12, 14–16]. Clinical trials have also reported positive outcomes, demonstrating significant antidepressant effects of PA [12]. However, the effectiveness of interventions remains inconsistent, particularly regarding fostering sustainable behavioral changes over the short and long term [11, 17, 18]. Notably, only a fraction of interventions has successfully achieved meaningful behavior change, which may be attributed to the diversity of intervention designs, variations in study quality, and differing methods for evaluating and analyzing the variables of interest [17].

Furthermore, the literature regarding the long-term effects of PA among distressed individuals remains inconsistent, potentially due to lower rates of adoption and long-term maintenance compared to the general population. Contributing factors include self-stigma, low motivation, and lack of self-confidence, which are often exacerbated by psychiatric symptoms, medication side effects, and co-occurring physical health conditions - all of which hinder sustained engagement in PA programs [19]. While short-term benefits, such as reductions in depressive symptoms [4, 6, 8] and increased PA levels [12], are better documented, findings remain inconclusive for certain populations. Therefore, investigating long-term outcomes, assessed months after the completion of interventions, is crucial to bridging knowledge gaps regarding the sustainability of behavior change and the optimization of mental health benefits.

However, research indicates a lack of consensus on the most appropriate indicators of PA for mental health benefits [20]. For instance, compliance with PA recommendations and higher levels of active behavior are widely recognized as protective factors against depression [13], although other forms of PA have also reduced depressive symptoms [21]. Moreover, while moderate- to vigorous-intensity PA appears to be more effective in alleviating symptoms [12], prescribed intensity levels may negatively impact participation to programs, highlighting the importance of addressing individual needs and preferences [22, 23]. Studies suggest that both muscle-strengthening [24] and aerobic activities [12] can improve depressive symptoms, although direct comparisons between these two types of activity are lacking. Specific practices, such as outdoor walking or running, cycling, and team sports, have also been associated with lower odds of depression [25]. Also, factors such as enjoyment, mastery of activity-related skills, motivation, autonomy, social interaction, and a sense of belonging [26] may influence the relationship between PA and mental health [20]. These elements are often more evident in leisure and commuting contexts, which have shown more promising effects on depressive symptoms compared to labor-related PA [27, 28].

Additionally, given the low adherence of this population to PA programs [22, 23] and the multifactorial nature of this behavior [16], it is crucial to identify which subgroups are most responsive to these intervention strategies. Despite this need, subgroup analyses remain underexplored [13, 29]. In a systematic review, Schuch and colleagues [29] identified that certain clinical (e.g., somatic symptoms, state anxiety, and global functioning), psychological (e.g., life satisfaction and self-esteem), social (e.g., social support and marital status), and biological (e.g., BDNF and TNF-α) characteristics may moderate the effects of PA on depression. However, the authors caution that the limited number of studies and the strength of the available evidence must be considered [29], underscoring the need for further research in this area. Although sex and age also appear to influence the relationship between PA and depression, these variables have not been identified as moderators [29]. Nevertheless, they warrant further investigation, as they remain largely underexplored [13].

Given the aforementioned, this study aimed to assess the short- (post-intervention) and long-term (follow-up) effects of a PA intervention on various indicators of this behavior and to explore potential moderators (sex and age) in adults with moderate to severe depressive symptoms.

## Methods

### Study design

The *Vincular* Project is a randomized, controlled, assessor-blinded clinical trial with two parallel groups (control and intervention) conducted in accordance with the CONSORT guidelines (Additional file 1) [30]. The intervention components were also reported following the TIDieR checklist (Additional file 2). The research protocol adheres to the Declaration of Helsinki, was approved by the Human Research Ethics Committee (CAAE: 60378122.1.0000.012), and all participants provided written informed consent prior to enrollment. The study protocol was registered in the Brazilian Clinical Trials Registry (ReBEC) under the code RBR-7466htj. The *Vincular* Project protocol study is in the final stages of publication. Further methodological details can be found in it [31].

### Protocol deviations/changes

The study protocol was submitted to the local Ethics Committee and to the ReBEC during the same period, prior to the initiation of any study procedures. Ethical approval was granted in September 2022, whereas registration in ReBEC was finalized in April 2023. The delay in trial registration was due to iterative requests from the registry team for formatting adjustments, despite the protocol content remaining unchanged. Importantly, no modifications to the study protocol were made after trial commencement. The intervention design and delivery, eligibility criteria, sample size, outcomes, and planned analyses were implemented as originally specified. Therefore, no protocol amendments requiring additional ethics approval were necessary.

### Subjects

The main eligibility criterion was presenting moderate to severe depressive symptoms, based on a score ≥ 9 on the Patient Health Questionnaire (PHQ-9) [32]. Additional criteria included residing in Florianópolis and metropolitan cities, being between 20 and 59 years old, owning a smartphone, having internet access, being available during scheduled meeting times, and not being pregnant or in the postpartum period. Participants were excluded if they required specialized psychiatric treatment for severe mental illnesses (e.g., schizophrenia), based on self-reported diagnosis; if they were at risk of suicide (indicating “several days,” “more than half the days,” or “nearly every day” for the suicide risk question on the PHQ-9); or if they had physical illnesses or limitations that prevented participation in the study. It is noteworthy that psychoeducational materials on mental health were provided, and referrals were made for individuals who were not included in the study and those identified as being at risk of suicide.

### Care providers

Three exercise specialists with expertise in health-related PA were responsible for standardizing the intervention procedures. Additionally, external guests with training in various fields contributed to the proposed activities and sessions.

### Recruitment, screening and selection

Participants were recruited through social media campaigns, the distribution of letters and pamphlets, and advertisements in local newspapers and podcasts between November 2022 and January 2023. Interested individuals signed an informed consent form and completed an initial screening questionnaire, both conducted online. Those who did not meet the eligibility criteria were excluded from the study. While direct referrals to professionals were not made, participants were advised to seek appropriate care, and a list of options was provided. Eligible participants were invited to an orientation meeting for further clarification, completion of additional assessments, and distribution of accelerometers.

### Randomization and blinding

The sample size was calculated using G*Power software (version 3.1.9.2), considering a medium effect size of 0.25 for depressive symptoms (the primary outcome of the *Vincular* Project), a statistical power of 80%, and a 5% significance level for two-tailed tests (ANOVA for repeated measures with three time points and two groups). This calculation yielded a sample size of 28 individuals. To account for potential dropout of up to 50% of participants, the required sample size was doubled, resulting in a total of 56 participants.

Randomization was performed using the online tool randomizar.org, with stratification based on sex, age, and PHQ-9 scores. The allocation sequence was generated by a researcher not directly involved in the study, and the allocation was concealed from the research staff responsible for data collection throughout the trial (undergraduate and graduate students who were previously trained and were not involved in other aspects of the study). Outcome assessors remained unaware of participants’ group allocation during all assessment procedures. Data analysis was conducted using anonymized datasets, without access to identifying information or group labels. However, due to the specific nature of the intervention groups, blinding was not feasible for participants and care providers.

### Experimental procedure

The intervention was conducted twice a week, with each session lasting 90 min, over a period of 16 weeks, starting in February 2023. Approximately 80% of the sessions were held in person, at locations selected according to the topic addressed on each day, while 20% took place online via video calls. All activities were grounded in Self-Determination Theory, which facilitates understanding of the different types of motivation that influence individuals to adopt or refrain from certain behaviors^33^. According to this theory, the more autonomous (or self-determined) an individual is, the more adaptive health outcomes are likely to be, thereby facilitating the adoption and maintenance of desired behaviors [33, 34].

The intervention aimed to increase participants’ awareness of their living and health conditions, focusing primarily on PA and its relationship with depressive symptoms. Sessions included lectures on adopting and maintaining healthy habits, distribution of educational materials related to depressive symptoms, PA, and other health-related behaviors (e.g., sleep and sedentary behavior), group discussions, and practical activities. These practical activities often involved family and friends and encompassed a variety of PA, such as yoga, capoeira, exergames, bike rides, walking pets, weight training, functional training, workplace gymnastics, and group exercise classes. Activities were implemented across different contexts, including leisure, commuting, and work.

The intervention program was structured into four blocks, each lasting four weeks and comprising eight sessions. These blocks were organized as follows: (a) contextualization: with theoretical sessions that explored the guiding themes of the *Vincular* Project and facilitated participant integration; (b) adherence: with general and specific guidelines on managing 24-hour movement behaviors in daily life; (c) experimentation: with sessions involving diverse PA practices; (d) maintenance: with practical sessions complemented by discussions on strategies to sustain healthy habits over the long term and without supervision, considering the end of the intervention period. Table 1 provides an overview of the activity schedule and the strategies employed to satisfy basic psychological needs.

**Table 1:** Intervention activities schedule from *Vincular* Project

Themes	Description and dynamics	Strategies for psychological needs
1. Introductory lesson □	• Presentation of the program schedule • Presentation: the speaker throws a ball to a random person, who has to introduce themselves. The person who has just introduced themselves throws the ball to someone else, and so on	*Relatedness*: - Considering knowing the participants’ life story - Engaging actively in activities, including doing and playing together - Valuing group activities
2. Depressive symptoms □	• Theoretical class on depressive symptoms • Discussion for coping possibilities • “Breaking sedentary behavior”. Participants stand in a circle. A rope is placed in the middle of the circle and some depressive symptoms are mentioned, as well as common situations among those who suffer from depressive symptoms. Those who perceive the symptoms and situations mentioned should approach the rope	*Autonomy*: - Reflecting on reasons for adhering to the programs of PA - Tracing short-term goals *Competence*: - Providing educational material containing multidisciplinary information pertinent to the PA and health relationship
3. Introduction to PA ○	• Theoretical class regarding the concept of PA, its benefits, types and domains, and its relationship with depression • “Do you practice PA?“. Participants are asked to vote on whether they do any PA. • “What are the benefits of PA?“. Through an online platform, participants must report the benefits they know about the regular practice of PA and these will appear in a “word cloud” format	*Autonomy*: - Ensuring that the discourse is not mandatory, that ensures choice about what to do, such as different types of exercises, about how to adapt the activities *Competence*: - Situations that culminate in gamified processes
4. Introduction to sedentary behavior and sleep ○	• Theoretical class on sedentary behavior and sleep • “What can we do to break sedentary behavior?“. Participants should list strategies or tips on how to reduce sedentary behavior • “How can we get a better quality of sleep?“. Participants should list strategies or tips related to sleep hygiene	*Autonomy*: - Ensuring that the discourse is not mandatory, that ensures choice about what to do, such as different types of exercises, about how to adapt the activities *Competence*: - Situations that culminate in gamified processes
5. Health-related physical fitness □	• Practice with different stations exploring the elements of health-related physical fitness: - Warm-up: “10 passes” - Cardiorespiratory fitness: running and jumping jacks - Strength: squat and push-up - Agility: “boss sent” - Dynamic balance and coordination - Static balance - Flexibility	*Autonomy*: - Adjusting the level of demand for the participants’ capacity to respond to the task *Competence*: - Adapting and building environmental alterations that ensure the beginning of the activity - Providing positive feedback to the class and individually, whether recognizing an effort of the participant or complimenting his attitude
6. Barriers and facilitators to PA ○	• Theoretical class with reflections on the barriers and facilitators to PA • Using the Mentimeter platform, identify the main barriers to PA perceived by the participants and discuss them, seeking to explore the frequency with which the barriers are perceived and the main reasons for this	*Competence*: - Different conditioning forms of achieving the goals *Relatedness*: - Encouraging the participant to invite friends and Family to carry out/participant in the programs of PA
7. Curiosities about practicing PA ○	• Question and answer class about PA • Interactive card-based dynamic	*Autonomy*: - Opting for language that ensures choice about what to do, such as different types of exercise, about how to adapt the activities *Competence*: - Providing educational material containing multidisciplinary information pertinent to the PA and health relationship
8. Lecture “Awaken the incredible in me” □	• Conversation • Dance • Emotional support • Dynamics in pairs	*Competence*: - Recognizing the improvement reached by the participant and remembering the “path” taken to reach it
9. Conducting the intervention □	• Meeting to discuss the experiences of the contextualization block • “Knot dynamics”. The participants stand in a circle and memorize who is on their left and right side. To the sound of music, they walk freely around the room. When the teacher asks them to, they must stop where they are and hold hands with the people next to them, but without moving. The aim is to undo the knot and return to the starting position	*Competence*: - Recognizing the stages for behavior chances of each participant *Relatedness*: - Encouraging the group`s cohesion in the activities and resolution of problems
10. Tools for 24-hour movement patterns ○	• Session to provide information on resources that help promote PA, reduce sedentary behavior and improve sleep quality • Practicing PA and meditation through YouTube channels	*Autonomy*: - Favoring that the participant has and perceives option of choice in the conduction of all stages relative to the program of PA *Relatedness*: - Encouraging the participant to invite friends and Family to carry out/participant in the programs of PA
11. Exergames □	• Practice with Xbox 360: Just Dance, Kinect Sports	*Relatedness*: - Engaging actively in activities, including doing and playing together
12. Lecture “Short and firm steps: an invitation to self-care with compassion” □	• Conversation • Presentation dynamics, with kindness and affection, considering other aspects than the professional life • Dream list, reflecting on their short-, medium- and long-term dreams	*Competence*: - Centering goals in the process - Valuing self-comparison as opposed to comparison with peers
13. Environment, PA and their possibilities □	• Practical class to explore and recognize a public space and discover the diversity of PA practices (collective vs. individual activities, outdoors vs. indoors, recreational vs. competitive, with vs. without supervision) • Recognizing space based on characteristics related to resources, conditions, access, aesthetics and safety • “Human tic-tac-toe” • “Playing with a hula hoop” • “Steal tail” • “Flag-picking” • Talking about collective vs. individual activities, outdoors vs. indoors, recreational vs. competitive, with vs. without supervision	*Autonomy*: - Recognizing the environmental potential for exercising by ensuring multiple potentials - Adapting and building environmental alterations that ensure the beginning of the activity - Considering participants’ reasons to exercise, such as preferences, expectations about health outcomes, and the cognitive and affective relationships established in the context of PA *Competence*: - Creates challenges to break the routine of the exercise sessions, whether for carrying out an exercise in a more challenging manner or carrying out activities in other environments
14. Capoeira □	• Practical initiation exercises • Activities with the basic elements and methodological aspects of capoeira teaching	*Competence*: - Creates challenges to break the routine of the exercise sessions, whether for carrying out an exercise in a more challenging manner or carrying out activities in other environments *Relatedness*: - Valuing group activities
15. Les Mills □	• Power jump • Bodyattack	*Autonomy*: - Favoring that the participant has and perceives option of choice in the conduction of all stages relative to the program of PA
16. Volleyball □	• Educational and warm-up exercises • Doubles exercises (set, bump, and ball control) and mini court exercises • Game	*Competence*: - Providing positive feedback to the class and individually, whether recognizing an effort of the participant or complimenting his attitude *Relatedness*: - Valuing group activities
17. Conducting the intervention □	• Meeting to discuss the experiences of the adherence block • “Compliments board”. Knowing that participants have difficulty identifying and recognizing their own qualities, each participant should praise their colleagues, creating a repertoire of positive characteristics of each one	*Autonomy*: - Raising the participants’ awareness about the need for advancing in the dynamics involved in the program of physical activities *Competence*: - Reflect with participants on aspects that still need improvement, visualize how to proceed/continue
18. Weight training □	• Resistance training class for the main muscle groups, using resources available in the gym (weights and machines) - Joint warm-up - Dumbbell bench press - Low row - Lateral raise - Dumbbell curl - Triceps on pulley - 45º leg press - Extension and flexor chair - Adductor and abductor chair - Calf raises	*Autonomy*: - Favoring that the participant has and perceives option of choice in the conduction of all stages relative to the program of PA *Relatedness*: - Engaging actively in activities, including doing and playing together
19. Functional training □	• Practice in circuit format focused on activities related to strength, control, stability and motor coordination: - Joint warm-up - Climber + jump on bench - Abdominal rowing - Support / push-up - Pull-up / inverted crucifix - Dips + lateral raises - Combined exercise - Plyometric - Abdominal plank - Coordination + squat - Juggling + jump rope	*Autonomy*: - Recognizing the environmental potential for exercising by ensuring multiple potentials *Competence*: - Different conditioning forms of achieving the goals
20. Dance □	• Dance initiation, including samba no pé, samba de gafieira, and zouk	*Competence*: - Creates challenges to break the routine of the exercise sessions, whether for carrying out an exercise in a more challenging manner or carrying out activities in other environments
21. Yoga □	• Beginners’ practice and breathing exercises	*Autonomy*: - Adjusting the level of demand for the participants’ capacity to respond to the task *Competence*: - Providing positive feedback to the class and individually, whether recognizing an effort of the participant or complimenting his attitude
22. Slackline □	• Stretching exercises • Balance exercises • Slackline practice	*Autonomy*: - Rationalizing the role of PA for reasons such as building a sense of friendship, improvement of skills, energy gain, and a better lifestyle
23. Cycling tour □	• Route from the university to the seafront	*Relatedness*: - Valuing group activities
24. Sand sports (beach tennis) □	• Warm-up • Ball handling drills • Service drills • Moving drills • Mini game	*Autonomy*: - Adjusting the level of demand for the participants’ capacity to respond to the task *Relatedness*: - Creating groups in virtual environments for experience exchange, facilitating the communication between participants
25. Conducting the intervention □	• Meeting to discuss the experiences of the experimentation block • Message from participants’ families encouraging them to continue with the project	*Autonomy*: - Assessing and reassessing long-term aims - Recognizing internal reasons relative to PA, such as satisfaction and pleasure *Competence*: - Recognizing the improvement reached by the participant and remembering the “path” taken to reach it
26. Pet day □	• Walk with participants’ pets	*Relatedness*: - Encouraging the participant to invite friends and family to carry out/participant in the programs of PA - Valuing group activities
27. Workers’ quality of life and active ageing ○	• Conversation round	*Autonomy*: - Considering participants’ reasons to exercise, such as preferences, expectations about health outcomes, and the cognitive and affective relationships established in the context of PA
28. Labour gymnastics □	• Stretching practice • Group dynamics • Self-massage and guided relaxation	*Autonomy*: - Opting for language that ensures choice about what to do, such as different types of exercise, about how to adapt the activities
29. Lecture “Quality of life and eating behavior: learning to deal with emotional eating” ○	• Chat session, with moments of reflection and awareness on the subject of quality of life and eating behavior	*Autonomy*: - Favoring that the participant has and perceives option of choice in the conduction of all stages relative to the program of PA *Competence*: - Providing educational material containing multidisciplinary information pertinent to the PA and health relationship
30. Gymnastics for everyone □	• Beginners’ practices	*Autonomy*: - Adjusting the level of demand for the participants’ capacity to respond to the task *Competence*: - Providing positive feedback to the class and individually, whether recognizing an effort of the participant or complimenting his attitude
31. Challenge day □	• Collective and competitive activities through games with family and friends - “Equal numbers” - “Three-legged race” - “Egg on spoon race” - “Unscrambling the letters” - “Emperor penguin” - “Bridge with hula hoops” - “Target shooting” + “balloon popping”	*Competence*: - Different conditioning forms of achieving the goals *Relatedness*: - Creating situations that essentially culminate in the participation of family and friends in activities - Encouraging the group`s cohesion in the activities and resolution of problems
32. Project closing trail □	• Trail • Reflections on the trajectory of the intervention and future prospects	*Autonomy*: - Rationalizing the role of PA for reasons such as building a sense of friendship, improvement of skills, energy gain, and a better lifestyle *Competence*: - providing positive feedback to the class and individually, whether recognizing an effort of the participant or complimenting his attitude *Relatedness*: - Creating groups in virtual environments for experience exchange, facilitating the communication between participants

Meetings 1 to 8 = contextualization block; Meetings 9 to 16 = adherence block; Meetings 17 to 24 = experimentation block; Meetings 25 to 32 = maintenance block; □ = face-to-face meeting; ○ = online meeting; PA = physical activity

The intervention can be characterized as an unconventional PA program. In this context, “unconventional” refers not to the activities themselves, but to the structure of the program, which differs from traditional exercise-based interventions that typically prescribe specific modalities, intensities, and progression based on the FITT principle. Instead, this intervention emphasized exposure to a wide range of PA practices across different contexts, combined with psychoeducational components aimed at increasing awareness and supporting self-regulation.

Accordingly, exercise intensity was not formally prescribed or monitored using physiological indicators (e.g., heart rate, METs, or ratings of perceived exertion). Participants were encouraged to engage at a self-selected level of effort according to their individual capacities, with adaptations made when necessary to ensure safety and inclusiveness.

To ensure consistency across sessions, all activities were delivered by trained facilitators following a structured program based on Self-Determination Theory, organized into predefined thematic blocks. This structure provided a common framework while allowing flexibility in the types of activities performed, aiming to enhance engagement and help participants identify personally meaningful practices that could be sustained beyond the intervention period.

In parallel, the control group was instructed to maintain their usual activities. To minimize dropout and maintain engagement, participants received telephone messages every two weeks. These messages included psychoeducational materials on mental health, as well as general guidance on engaging in PA, reducing sedentary behavior, and improving sleep quality, and the content was based on established national and international guidelines (e.g., World Health Organization and national health agencies). This approach can be characterized as an active informational comparison condition, intended to maintain participant engagement without providing a structured behavioral intervention. Participants were also informed that, if the intervention proved effective, they would be offered the program after the study concluded. Additional details and examples of the materials are provided in previously published article [35].

### Variables and instruments

Assessments were conducted at three time points: (a) baseline (January 2023); (b) immediately after the intervention (May 2023); and (c) follow-up, six-months post-intervention (November 2023). This study represents a secondary analysis of a broader randomized controlled trial in which depressive symptoms were the primary outcome and PA was assessed as a secondary outcome.

Active behavior – used, in the present study, to refer to movement-related behaviors involving bodily activity across different domains and contexts of daily life, in contrast to sedentary behavior – was assessed through both self-reported and objectively measured using accelerometers. Part of the questionnaire used by the Brazilian survey known as “Surveillance of Risk and Protective Factors for Chronic Diseases by Telephone Survey” (VIGITEL) was used to assess PA across different domains: commuting, domestic, leisure, and work. This instrument was developed based on tools utilized in systems monitoring risk factors for chronic diseases [36]. Additionally, the Simple Physical Activity Questionnaire (SIMPAQ) was employed to provide a validated measure for individuals living with mental illnesses. The SIMPAQ evaluates PA and sedentary behavior by capturing time spent in the following contexts: (a) in bed; (b) sedentary behavior; (c) walking for exercise, recreation, or commuting; (d) exercising; and (e) other activities, such as work-related tasks or household chores [37]. For this study, only activities classified as active behavior (walking, exercising, and other activities) were analyzed.

Objectively-assessed PA were obtained using ActiGraph GT3X + and wGT3X+ accelerometers (ActiGraph Corporation, Pensacola, Florida, USA) worn on the non-dominant wrist for seven consecutive days. Participants followed to a 24-hour usage protocol, removing the device only for activities involving water submersion. Accelerometer devices were initialized with a data acquisition frequency set at 30 Hz using the ActiLife software (version 6.8.11 for Windows). The same software was used to download the raw .gt3x files and convert them to .csv format for data processing using the GGIR package (version 2.9.5) in R. Given that a 24-hour accelerometer wear protocol was used, the data were processed to capture 24-hour movement behaviors by separating waking and sleeping periods. The data-processing pipeline included a self-calibration procedure for the raw acceleration data, which were converted to the ENMO (Euclidean Norm Minus One) metric, expressed in milligravitational units (mg), using 5-second epochs. Non-wear time was assessed using a validated algorithm [38]. Data were considered valid if participants provided at least three days with a minimum of 16 h of wear time per day, including two weekdays and one weekend day. Time gaps identified as non-wear periods within each 24-hour interval meeting the validation criteria (≥ 16 h) were imputed at the raw-data level using average values across valid days. Active behavior was classified according to the cut-off points proposed by Hildebrand et al.‘s [39] cut-off points as follows: (a) light PA (35.6–201.4 mg); (b) moderate to vigorous PA (≥ 201.4 mg); and (c) total PA (≥ 35.6 mg). The sleep window was identified using a heuristic algorithm that examined the distribution of changes in the z-angle [40]. This algorithm distinguishes sleep from other inactivity periods by identifying the longest sustained period of inactivity with the fewest interruptions within a 24-hour window A measurement day was defined as the interval between wake-up times. To isolate waking behaviors, epochs classified as inactive (i.e., below the light PA threshold), as well as the sleep window, were excluded from the measurement day. Consequently, PA was derived exclusively from epochs occurring during wake periods. Finally, time spent in PA was weighted according to weekdays and weekends using a 5:2 ratio and averaged across valid days for each participant.

It is important to note that for self-reported measures, different recall periods were considered according to the instrument. The VIGITEL questionnaire assesses habitual patterns of PA maintained over at least the previous three months, whereas the SIMPAQ captures PA based on the previous seven days at each assessment point. For objectively measured PA, participants were instructed to wear the accelerometer for seven consecutive days immediately following each assessment point. Therefore, accelerometer data collected at post-intervention reflect PA during the week after completion of the intervention, rather than behavior during the active intervention period.

### Safety procedures and adverse events

Given the nature of the intervention and the clinical condition of the sample, safety procedures were implemented to protect participants. First, the research team’s contact information (telephone and email) was provided in the informed consent form and all study materials. Participants were encouraged to contact the team with any concerns or questions during the intervention. All messages and emails were responded to within 24 h, and face-to-face conversations were also conducted to provide individualized support and ensure participant safety. Additionally, if a participant missed two consecutive sessions, the team reached out to check on their well-being. In cases of withdrawal, the reasons for discontinuation were explored and documented.

Adverse events were monitored through passive surveillance during the intervention and follow-up periods, based on participant self-report and direct communication with the research team. For the purposes of this study, an adverse event was defined as any unfavorable physical or psychological symptom or condition occurring during the study period, whether or not considered related to the intervention.

### Statistical analysis

Descriptive statistics were calculated for all variables. Baseline differences between groups were assessed using chi-square tests for categorical variables and independent-samples t-tests for continuous variables.

**Fig. 1:**
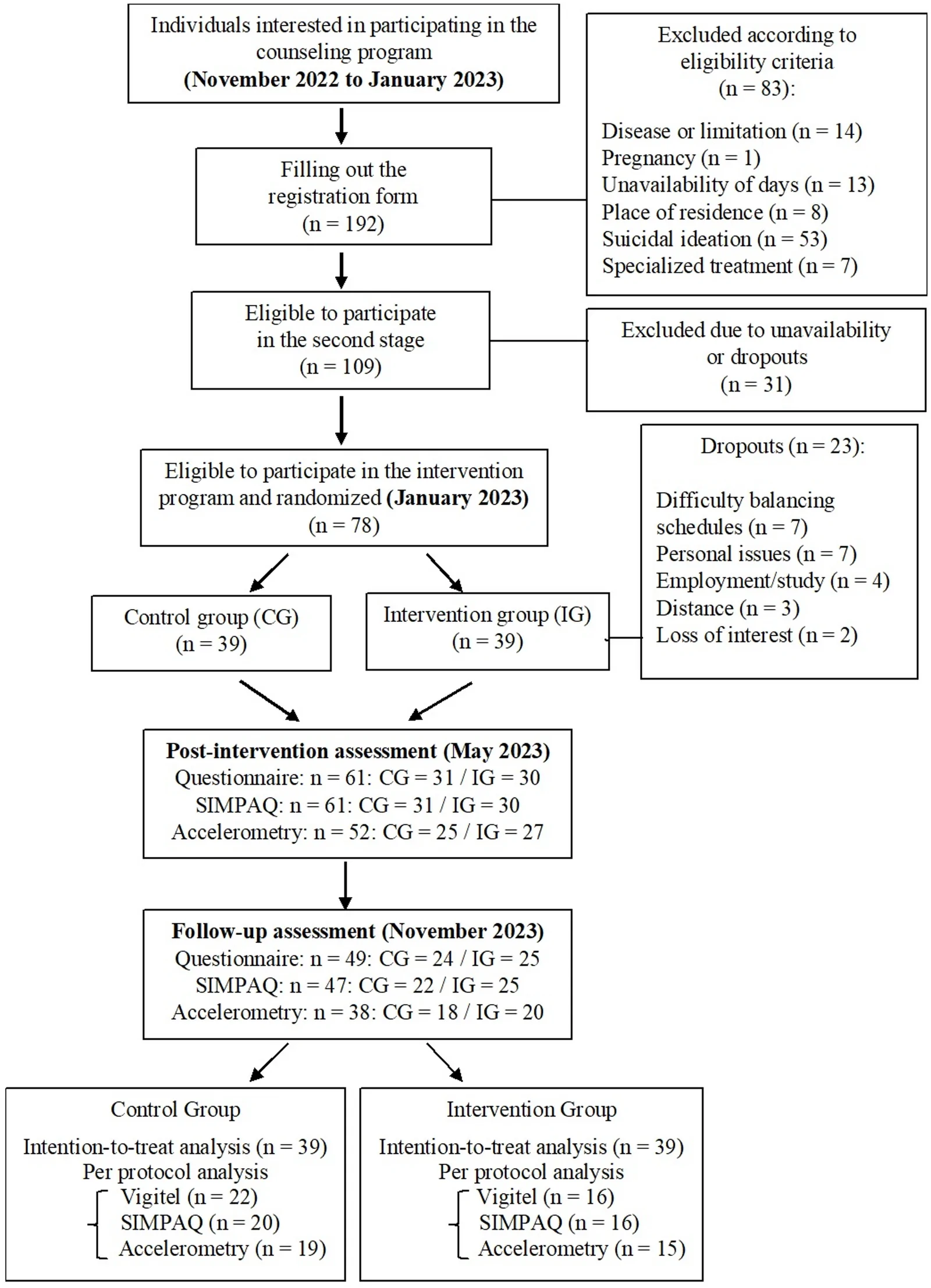
Flow diagram. Florianópolis, SC, Brazil, 2023. Notes: Vigitel = “Surveillance of risk and protective factors for chronic diseases by telephone survey” instrument; SIMPAQ = Simple Physical Activity Questionnaire

To evaluate the short- and long-term effects of the intervention on various PA indicators, both per-protocol and intention-to-treat analyses were conducted; however, intention-to-treat analyses were considered the primary basis for inference. In the per-protocol analysis, only participants who attended the intervention activities until the final session, complied with the protocol (with at least 50% overall attendance and in each of the four intervention blocks), and were properly assessed at all three time points (baseline, post-intervention, and follow-up) were included. In the intention-to-treat analysis, all randomized participants were analyzed according to their original allocation, regardless of their adherence to the intervention, any protocol deviations, or other issues that may have occurred after randomization. Missing data from the post-intervention and follow-up collections were handled using multiple imputation. The extent and pattern of missing data by group, time point, and outcome are presented in Fig. 1. Multiple imputation was conducted using the fully conditional specification (FCS) method, with predictive mean matching (PMM) applied for continuous variables. The imputation models included depressive symptoms (PHQ-9), sex, age, and time-varying PA indicators as predictors. Baseline values had no missing data. To account for the interaction effects between repeated measurements (i.e., baseline, post-intervention and follow-up) and conditions (i.e., control and intervention), imputations were performed separately for each condition. A total of five imputed datasets were generated for each condition, and the results were pooled according to Rubin’s rules. Convergence of the imputation models was assessed through inspection of the imputation process, and no apparent irregularities were identified. Baseline comparisons between completers and non-completers were conducted to characterize potential differences related to attrition. Although the missing at random (MAR) assumption cannot be verified empirically, depressive symptom severity, sex, age, and time-varying physical activity indicators were included in the imputation models because these variables were considered potentially related to both attrition and study outcomes. Including observed predictors of missingness may improve the plausibility of the MAR assumption and reduce potential bias associated with incomplete data.

For comparisons between periods and groups, Generalized Estimating Equations (GEE) were used, with Bonferroni’s post-hoc test applied. The significance level was set at *p* ≤ 0.05. Effect estimates are presented as within-group mean changes from baseline to post-intervention and follow-up (Δ), and as between-group differences in change (difference-in-differences), with corresponding 95% confidence intervals (95% CI). Exploratory analyses were conducted to examine potential moderators of the intervention effect, testing three-way interaction terms (group*time*moderator) and considering sex (men and women) and age (20 to 36 years and 37 to 59 years) as moderators. These analyses were not prespecified in the trial protocol or registration and should be interpreted as hypothesis-generating. Additional exploratory analyses, including only individuals who did not meet the baseline PA recommendations, were also performed and are available in Additional file 3. In these analyses, only the effect of the intervention on leisure-time PA levels was investigated. Simple main effects were examined for significant three-way interaction terms with *p* < 0.10. All analyses were performed using the Statistical Package for the Social Sciences (SPSS, version 23.0).

## Results

A total of 78 individuals were randomized into the control and intervention groups (39 adults in each group). At the end of the intervention, 61 participants completed the questionnaires, and 52 complied to the accelerometer protocol, providing valid data. At the follow-up assessment, the number of participants completing the questionnaires and accelerometer protocol decreased to 49 and 38, respectively (Fig. 1). The dropout rate for the intervention was 59%. Regarding intervention attendance, the overall compliance rate (average number of confirmed attendances per participant divided by the total number of sessions) among all participants allocated to the intervention group (*n* = 39) was 34.8%, while among those who completed the intervention (*n* = 16) it was 73.1%. For the intention-to-treat analyses, missing data were handled using multiple imputation. At post-intervention, 17 questionnaire/SIMPAQ observations (8 in the control group and 9 in the intervention group) and 26 accelerometry observations (14 and 12, respectively) were imputed. At follow-up, the number of imputations increased to 29 for questionnaire data (15 and 14), 31 for SIMPAQ (17 and 14), and 40 for accelerometry (21 and 19) in the control and intervention groups, respectively.

No adverse events were reported or observed throughout the intervention. Furthermore, no significant differences were found between the control and intervention groups at baseline in terms of sociodemographic variables (sex [*p* = 1.000], age [*p* = 0.939], skin color [*p* = 0.620], marital status [*p* = 0.802], and education [*p* = 0.648]), clinical variables (depressive symptom score [*p* = 0.837], mental illness diagnosis [*p* = 0.479], depression alone [*p* = 1.000], medication use for depression [*p* = 0.810], psychotherapy [*p* = 0.129], and family history of depression [*p* = 0.849]), and related to PA indicators (light PA [*p* = 0.789], moderate-to-vigorous PA [*p* = 0.819], total PA measured by accelerometry [*p* = 0.754], walking [*p* = 0.274], physical exercises [*p* = 0.090], other activities [*p* = 0.384], leisure PA [*p* = 0.201], commuting PA [*p* = 0.144], household PA [*p* = 0.249], and work PA [*p* = 0.533]). However, the level of total PA, measured by questionnaire, was higher in the intervention group compared to the control group (*p* = 0.039). To further explore potential sources of attrition, Additional file 4 presents baseline characteristics comparing participants who completed the intervention protocol and those who did not, both within the intervention group and across the total sample. The only variable that differed between groups was the PHQ-9 total score, with participants who completed the intervention presenting lower depressive symptom severity at baseline compared to those who did not complete the protocol.

Per-protocol analyses (Table 2) indicate a significant group by time interaction for leisure-time PA (*p* = 0.004), where the intervention condition increased from 135.0 (± 44.92) to 262.8 (± 40.35) min/week versus an increase from 115.5 (± 25.07) to 123.6 (± 25.69) min/week in controls from the baseline to post intervention (diff-in-diff = 119.6; 95% CI: -190.1 to -49.2). Group by interactions for all other PA outcomes were not significant.

**Table 2:** Per-protocol analysis of the effects and moderators of an intervention on physical activity indicators

Indicators	Baseline	Post-intervention	Follow-up	Δ (95% CI)		*p*-value				
	x̅ (± se)	x̅ (± se)	x̅ (± se)	Δ#	Δ$	g	t	g*t	g*t*sex	g*t*age
Accelerometry data (minutes/day): *n* = 34; CG: *n* = 19; IG: *n* = 15										
Light PA						0.218	0.598	0.146	0.359	0.259
CG	158.0 (± 8.40)	163.3 (± 8.98)	162.5 (± 9.43)	5.3 (-10.0 to 20.6)	4.5 (-9.7 to 18.8)					
IG	155.7 (± 10.18)	139.1 (± 11.23)	142.4 (± 11.33)	-16.6 (-32.3 to -0.9)	-13.3 (-32.3 to 5.7)					
Diff-in-Diff	---	---	---	-21.9 (-0.1 to 43.7)	-17.8 (-6.0 to 41.5)					
MVPA						0.086	0.174	0.363	0.295	**0.046**
CG	111.3 (± 11.81)	106.4 (± 10.59)	107.1 (± 13.33)	-4.9 (-18.6 to 8.8)	-4.2 (-17.4 to 9.0)					
IG	106.0 (± 20.09)	70.1 (± 5.88)	76.6 (± 6.23)	-35.9 (-76.4 to 4.6)	-29.4 (-66.2 to 7.5)					
Diff-in-Diff	---	---	---	-31.0 (-11.8 to 73.7)	-25.2 (-14.0 to 64.3)					
Total PA						0.090	0.112	0.105	0.636	**0.049**
CG	269.3 (± 18.28)	269.7 (± 17.39)	269.6 (± 21.48)	0.4 (-27.3 to 28.0)	0.3 (-25.2 to 25.8)					
IG	261.6 (± 22.38)	209.0 (± 16.08)	219.0 (± 16.17)	-52.5 (-94.2 to -10.7)	-42.6 (-78.8 to -6.5)					
Diff-in-Diff	---	---	---	-52.9 (2.7 to 102.9)	-42.9 (-1.3 to 87.2)					

Simple Physical Activity Questionnaire (SIMPAQ) (minutes/week): *n* = 36; CG: *n* = 20; IG: *n* = 16										
Walking						0.923	**0.005**	0.383	0.581	**0.026**
CG	34.4 (± 10.70)¨	58.2 (± 17.65)	87.7 (± 24.25)¨	23.9 (1.3 to 46.4)	53.3 (13.9 to 92.7)					
IG	49.2 (± 12.56)	71.0 (± 28.55)	66.2 (± 16.14)	21.8 (-23.2 to 66.8)	17.1 (-16.1 to 50.2)					
Diff-in-Diff	---	---	---	-2.1 (-48.3 to 52.3)	-36.2 (-15.3 to 87.7)					
Physical exercises						**0.030**	0.553	0.890	0.104	**0.005**
CG	15.6 (± 4.13)	14.3 (± 3.78)	19.8 (± 6.09)	-1.4 (-10.8 to 8.0)	4.2 (-10.0 to 18.3)					
IG	32.1 (± 8.89)	34.8 (± 8.69)	38.9 (± 9.86)	2.8 (-11.2 to 16.7)	6.8 (-8.4 to 22.1)					
Diff-in-Diff	---	---	---	4.2 (-20.9 to 12.7)	2.6 (-23.5 to 18.2)					
Other activities						0.945	0.135	0.253	**0.071**	0.680
CG	153.8 (± 25.51)	223.5 (± 39.88)	157.5 (± 28.86)	69.8 (-23.2 to 162.7)	3.8 (-60.9 to 68.4)					
IG	149.7 (± 19.90)	182.5 (± 31.47)	209.6 (± 44.16)	32.8 (-9.8 to 75.4)	59.9 (-22.6 to 142.3)					
Diff-in-Diff	---	---	---	-37.0 (-65.3 to 139.2)	56.1 (-160.9 to 48.6)					

Vigitel Questionnaire (minutes/week): *n* = 38; CG: *n* = 22; IG: *n* = 16										
Leisure						0.062	**0.001**	**0.004**	**0.056**	0.213
CG	115.5 (± 25.07)	123.6 (± 25.69)&	115.0 (± 31.98)	8.2 (-41.1 to 57.4)	-0.5 (-78.0 to 77.0)					
IG	135.0 (± 44.92)$	262.8 (± 40.35)&$	218.1 (± 55.35)	127.8 (77.4 to 178.2)	83.1 (3.1 to 163.1)					
Diff-in-Diff	---	---	---	119.6 (-190.1 to -49.2)	83.6 (-195.0 to 27.8)					
Commuting						0.249	0.619	0.899	**0.036**	0.193
CG	72.7 (± 20.64)	86.1 (± 21.27)	72.7 (± 22.78)	13.4 (-35.4 to 62.2)	-0.0 (-59.3 to 59.3)					
IG	131.6 (± 61.17)	150.0 (± 58.59)	114.7 (± 34.83)	18.4 (-84.5 to 121.4)	-16.9 (-107.3 to 73.6)					
Diff-in-Diff	---	---	---	5.0 (-118.9 to 108.9)	-16.9 (-91.2 to 125.0)					
Household						0.197	0.758	0.158	0.599	0.278
CG	163.0 (± 39.47)	151.8 (± 35.11)	181.8 (± 43.44)	-11.1 (-33.3 to 11.0)	18.9 (-16.5 to 54.2)					
IG	245.6 (± 57.32)	301.9 (± 103.80)	228.8 (± 63.63)	56.3 (-152.5 to 265.0)	-16.9 (-169.6 to 135.8)					
Diff-in-Diff	---	---	---	67.4 (-277.3 to 142.5)	-35.8 (-121.0 to 192.5)					
Work						0.791	0.621	0.377	**0.048**	0.107
CG	166.4 (± 109.77)	115.9 (± 63.91)	130.9 (± 104.97)	-50.5 (-273.7 to 172.8)	-35.5 (-295.8 to 224.8)					
IG	0	131.3 (± 87.82)	206.3 (± 121.39)	131.3 (-40.9 to 303.4)	206.3 (-31.7 to 444.2)					
Diff-in-Diff	---	---	---	181.8 (-463.6 to 100.2)	241.8 (-594.4 to 111.0)					
Total PA						0.273	0.487	0.334	**0.066**	0.259
CG	517.5 (± 148.35)	477.5 (± 73.45)	500.5 (± 109.76)	-40.0 (-306.3 to 226.3)	-17.0 (-298.5 to 264.4)					
IG	512.2 (± 110.63)	845.9 (± 235.21)	767.8 (± 214.54)	333.7 (-84.8 to 752.3)	255.6 (-46.5 to 557.7)					
Diff-in-Diff	---	---	---	373.7 (-869.8 to 122.3)	272.6 (-685.5 to 140.2)					

Bold values indicate statistically significant results. The significance level was set at *p* ≤ 0.05. Simple main effects were examined for significant three-way interaction terms with *p* < 0.10

*PA *Physical activity, *MVPA *Moderate-to-vigorous physical activity, *CG* Control group, *IG *Intervention group

x̅ = mean; ± se = standard error; Δ# = post-intervention mean − baseline mean; Δ = difference between post-intervention and baseline; & = difference between intervention group and control group

Exploratory analyses suggested that sex may have moderated the effects of the intervention on several PA indicators, including other activities, leisure-time PA, commuting PA, work-related PA, and total PA (Table 2). The intervention effects on leisure and total PA appeared more pronounced among women than men. For leisure PA, the between-group difference in change (diff-in-diff) from baseline to post-intervention was 142.1 min/week (95% CI: -25.2 to 309.5) in women versus 70.3 min/week (95% CI: -209.0 to 349.7) in men; from baseline to follow-up, the diff-in-diff was 167.3 min/week (95% CI: -17.6 to 352.2) in women and − 107.9 min/week (95% CI: -464.4 to 248.7) in men. For total PA, the post-intervention diff-in-diff was 563.5 min/week (95% CI: -268.5 to 1395.5) in women and − 73.7 min/week (95% CI: -609.5 to 462.2) in men; at follow-up, the diff-in-diff remained large for women (513.8 min/week; 95% CI: -272.2 to 1299.9) and negative for men (–264.5 min/week; 95%CI: -1017.1 to 488.1). For commuting and work PA, no clear pattern of sex differences was observed (Table 3).

**Table 3:** Effects of an intervention on physical activity indicators according to sex (per-protocol analysis)

Men						Women					
Indicators	Baseline	Post-intervention	Follow-up	Δ (95% CI)		Baseline		Post-intervention	Follow-up	Δ (95% CI)	
	x̅ (± se)	x̅ (± se)	x̅ (± se)	Δ#	Δ$	x̅ (± se)		x̅ (± se)	x̅ (± se)	Δ#	Δ$
Simple Physical Activity Questionnaire (SIMPAQ) (minutes/week): *n*= 36; CG: *n* = 20; IG: *n* = 16											
Other activities											
CG	122.5 (± 57.49)	240.0 (± 73.14)&%	156.4 (± 62.68)	117.5 (-82.2 to 317.2)	33.9 (-147.3 to 215.2)	167.1 (± 26.04)		216.4 (± 47.45)	158.1 (± 28.85)	49.3 (-60.8 to 159.4)	-9.1 (-88.2 to 70.1)
IG	105.0 (± 23.24)	86.0 (± 14.31)&	73.6 (± 31.74)%	-19.0 (-78.8 to 40.8)	-31.4 (-117.6 to 54.8)	170.0 (± 24.63)		226.4 (± 38.64)	271.4 (± 52.91)	56.4 (-37.8 to 150.6)	101.4 (-18.7 to 221.4)
Diff-in-Diff	---	---	---	-136.5 (-345.0 to 72.0)	-65.3 (-266.0 to 135.4)	---		---	---	7.1 (-137.8 to 152.0)	110.5 (-33.4 to 254.3)

Vigitel Questionnaire (minutes/week): *n* = 38; CG: *n* = 22; IG: *n* = 16											
Leisure											
CG	65.0 (± 34.03)	71.7 (± 37.15)	172.9 (± 76.55)	6.7 (-101.5 to 114.8)	107.9 (-69.9 to 285.6)	134.4 (± 30.72)		143.1 (± 31.10)	88.0 (± 27.78)%	8.8 (-79.7 to 97.2)	-46.4 (-130.3 to 37.6)
IG	168.0 (± 93.57)	245.0 (± 71.13)	168.0 (± 105.50)	77.0 (-180.6 to 334.6)	0.0 (-309.0 to 309.0)	120.0 (± 48.94)*		270.9 (± 48.79)%*	240.9 (± 63.49)	150.9 (8.8 to 293.0)	120.9 (-43.9 to 285.7)
Diff-in-Diff	---	---	---	70.3 (-209.0 to 349.7)	-107.9 (-464.4 to 248.7)	---		---	---	142.1 (-25.2 to 309.5)	167.3 (-17.6 to 352.2)
Commuting											
CG	141.7 (± 46.27)%	120.0 (± 43.78)	46.4 (± 24.96)	-21.7 (-158.4 to 115.1)	-95.2 (-207.8 to 17.3)	46.9 (± 18.73)		73.4 (± 23.43)	85.0 (± 30.80)	26.6 (-34.2 to 87.3)	38.1 (-35.0 to 111.2)
IG	58.0 (± 34.22)	15.0 (± 13.42)%	97.0 (± 69.33)	-43.0 (-123.6 to 37.6)	39.0 (-130.4 to 208.4)	165.0 (± 85.73)		211.4 (± 78.29)	122.7 (± 39.42)	46.4 (-192.3 to 285.0)	-42.3 (-236.2 to 151.7)
Diff-in-Diff	---	---	---	-21.3 (-180.1 to 137.4)	134.2 (-69.2 to 337.6)	---		---	---	19.8 (-226.5 to 266.1)	-80.4 (-287.7 to 126.9)
Work											
CG	150.0 (± 107.47)	275.0 (± 167.39)	402.9 (± 305.39)	125.0 (-302.1 to 552.1)	252.9 (-433.6 to 939.3)	172.5 (± 145.42)		56.2 (± 54.46)	4.0 (± 3.86)	-116.2 (-430.6 to 198.1)	-168.5 (-463.0 to 126.0)
IG	0	0	0	0.0 (0.0 to 0.0)	0.0 (0.0 to 0.0)	0		190.9 (± 123.62)	300.0 (± 169.18)	190.9 (-63.2 to 445.0)	300.0 (-47.8 to 647.8)
Diff-in-Diff	---	---	---	-125.0 (-552.1 to 302.1)	-252.9 (-939.3 to 433.6)	---		---	---	307.1 (-97.0 to 711.4)	468.5 (12.8 to 924.2)
Total PA											
CG	436.7 (± 108.45)	568.3 (± 179.34)	782.1 (± 112.09)	131.7 (-318.3 to 581.7)	345.5 (-313.1 to 1004.1)	547.8 (± 199.36)		443.4 (± 73.57)	369.0 (± 62.08)%	-104.4 (-534.5 to 325.8)	-178.8 (-601.6 to 243.9)
IG	274.0 (± 122.69)	332.0 (± 50.70)	355.0 (± 112.09)	58.0 (-232.9 to 348.9)	81.0 (-283.2 to 445.2)	620.5 (± 139.20)		1079.5 (± 317.24)%	955.5 (± 290.77)	459.1 (-253.1 to 1171.3)	335.0 (-327.7 to 997.7)
Diff-in-Diff	---	---	---	-73.7 (-609.5 to 462.2)	-264.5 (-1017.1 to 488.1)	---		---	---	563.5 (-268.5 to 1395.5)	513.8 (-272.2 to 1299.9)

*PA* Physical activity, *CG* Control group, *IG* Intervention group

x̅ = mean; ± se = standard error; Δ# = post-intervention mean − baseline mean; Δ$ = follow-up mean − baseline mean

Exploratory moderation analysis also revealed that the intervention had some effects among older adults (37–59 years) compared to younger adults (20–36 years), particularly for accelerometer-measured and structured PA (Table 4). For moderate-to-vigorous PA, the diff-in-diff from baseline to post-intervention was + 17.1 min/day (95% CI: -54.2 to 20.1) in older adults versus − 52.5 min/day (95% CI: -4.6 to 109.6) in younger adults; from baseline to follow-up, it was − 0.7 min/day (95% CI: -38.1 to 39.5) and − 40.2 min/day (95% CI: -10.9 to 91.4), respectively. For total PA, the post-intervention diff-in-diff was + 15.8 min/day (95% CI: -68.7 to 37.2) in older adults and − 82.1 min/day (95% CI: 20.2 to 144.0) in younger adults; at follow-up, it was − 10.2 min/day (95% CI: -62.2 to 82.6) and − 61.8 min/day (95% CI: 13.1 to 110.6), respectively. For physical exercises, older adults improved by 33.8 min/week (95% CI: -50.5 to -17.3) post-intervention and 20.2 min/week (95% CI: -44.0 to 3.5) at follow-up, while younger adults declined by 11.4 (95%CI: -20.3 to 33.2) and 6.6 min/week (95% CI: -21.1 to 34.1), respectively.

**Table 4:** Effects of an intervention on physical activity indicators according to age (per-protocol analysis)

	20 to 36 years					37 to 59 years					
Indicators	Baseline	Post-intervention	Follow-up	Δ (95% CI)		Baseline		Post-intervention	Follow-up	Δ (95% CI)	
	x̅ (± se)	x̅ (± se)	x̅ (± se)	Δ#	Δ$	x̅ (± se)		x̅ (± se)	x̅ (± se)	Δ#	Δ$
Accelerometry data (minutes/day): 34; CG:*n* = 19; IG:*n* = 15											
MVPA											
CG	112.0 (± 14.36)%	109.9 (± 12.87)&	104.5 (± 16.54)	-2.0 (-18.7 to 14.7)	-7.5 (-23.2 to 8.2)	108.7 (± 15.62)		93.0 (± 12.0)$	116.8 (± 11.44)$	-15.7 (-27.7 to -3.7)	8.1 (-9.2 to 25.5)
IG	118.9 (± 28.31)	64.4 (± 6.99)%&	71.2 (± 6.64)	-54.5 (-109.1 to 0.1)	-47.7 (-96.4 to 0.9)	80.0 (± 15.00)		81.4 (± 8.79)	87.4 (± 11.72)	1.4 (-33.8 to 36.5)	7.4 (-27.3 to 42.1)
Diff-in-Diff	---	---	---	-52.5 (-4.6 to 109.6)	-40.2 (-10.9 to 91.4)	---		---	---	17.1 (-54.2 to 20.1)	-0.7 (-38.1 to 39.5)
Total PA											
CG	262.0 (± 21.97)	268.1 (± 20.85)&	259.4 (± 26.22)	6.2 (-28.0 to 40.3)	-2.6 (-33.1 to 28.0)	296.8 (± 22.70)		275.4 (± 26.43)	307.9 (± 16.81)	-21.4 (-38.7 to -4.0)	11.1 (-26.9 to 49.1)
IG	261.6 (± 31.81)*¨	185.7 (± 18.74)&*	197.3 (± 17.46)¨	-75.9 (-127.5 to -24.3)	-64.4 (-102.4 to -26.3)	261.6 (± 21.48)		256.0 (± 16.19)	262.5 (± 23.81)	-5.6 (-55.6 to 44.4)	0.9 (-60.7 to 62.5)
Diff-in-Diff	---	---	---	-82.1 (20.2 to 144.0)	-61.8 (13.1 to 110.6)	---		---	---	15.8 (-68.7 to 37.2)	-10.2 (-62.2 to 82.6)

Simple Physical Activity Questionnaire (SIMPAQ) (minutes/week): *n* = 36; CG: *n* = 20; IG: *n* = 16											
Walking											
CG	36.1 (± 13.32)¨	73.0 (± 23.90)	107.8 (± 32.65)¨	36.9 (8.7 to 65.0)	71.7 (21.6 to 121.7)	30.4 (± 17.40)		23.8 (± 8.37)	40.7 (± 14.26)	-6.5 (-28.7 to 15.7)	10.4 (-33.6 to 54.3)
IG	39.9 (± 12.63)	51.0 (± 13.34)	46.4 (± 11.51)	11.1 (-17.0 to 39.3)	6.6 (-18.2 to 31.4)	64.6 (± 24.79)		104.3 (± 70.74)	99.2 (± 34.57)	39.7 (-69.3 to 148.7)	34.5 (-41.7 to 110.8)
Diff-in-Diff	---	---	---	-25.8 (-14.1 to 65.5)	-65.1 (9.2 to 120.9)	---		---	---	46.2 (-157.4 to 65.0)	24.2 (-112.2 to 63.8)
Physical exercises											
CG	13.0 (± 4.67)	17.3 (± 4.82)	20.6 (± 8.06)	4.3 (-6.9 to 15.6)	7.7 (-12.2 to 27.5)	21.8 (± 7.90)		7.2 (± 4.50)	17.9 (± 7.59)	-14.6 (-26.3 to -3.0)	-3.9 (-8.4 to 0.5)
IG	36.4 (± 12.64)	29.3 (± 10.50)	37.6 (± 11.49)	-7.1 (-25.7 to 11.5)	1.1 (-18.0 to 20.3)	24.8 (± 10.19)*		44.0 (± 14.46)*	41.1 (17.99) 41.1 (17.99)	19.2 (7.4 to 31.1)	16.3 (-7.0 to 39.6)
Diff-in-Diff	---	---	---	-11.4 (-20.3 to 33.2)	-6.6 (-21.1 to 34.1)	---		---	---	33.8 (-50.5 to -17.3)	20.2 (-44.0 to 3.5)

*MVPA* Moderate-to-vigorous physical activity, *PA* Physical activity, *CG* Control group, *IG* Intervention group

x̅ = mean; ± se = standard error; Δ# = post-intervention mean − baseline mean; Δ$ = follow-up mean − baseline mean

Intention-to-treat analyses can be found in Table 5. No significant differences were observed in group by time interaction. However, both physical exercise and total PA showed significant main effects for group and time, with higher averages in the intervention group and a significant increase from baseline to follow-up. While sex moderated some PA indicators (except moderate-to-vigorous PA, total PA, walking, commuting PA, and household PA – Table 6), age did not yield statistically significant results for the three-way interaction terms (group*time*moderator).

**Table 5:** Intention-to-treat analysis of the effects and moderators of an intervention on physical activity indicators

Indicators	Baseline	Post-intervention	Follow-up	Δ (95% CI)		*p*-value				
	x̅ (± se)	x̅ (± se)	x̅ (± se)	Δ#	Δ$	g	t	g*t	g*t*sex	g*t*age
Accelerometry data (minutes/day): *n* = 78; CG: *n* = 39; IG: *n* = 39										
Light PA						0.303	0.462	0.210	**0.015**	0.614
CG	159.8 (± 5.77)	161.0 (± 5.13)	165.0 (± 5.12)	1.2 (-10.5 to 13.0)	5.2 (-7.4 to 17.8)					
IG	162.1 (± 6.27)	150.9 (± 6.05)	152.9 (± 5.27)	-11.2 (-22.9 to 0.5)	-9.2 (-20.1 to 1.7)					
Diff-in-Diff	---	---	---	-12.4 (-4.1 to 29.0)	-14.4 (-2.2 to 31.1)					
MVPA						0.646	0.231	0.645	0.500	0.257
CG	95.8 (± 7.00)	97.6 (± 6.00)	102.0 (± 7.48)	1.8 (-8.2 to 11.9)	6.2 (-4.8 to 17.3)					
IG	98.4 (± 8.93)	89.6 (± 5.11)	97.2 (± 4.28)	-8.8 (-28.8 to 11.2)	-1.3 (-19.5 to 17.0)					
Diff-in-Diff	---	---	---	-10.6 (-11.7 to 33.0)	-7.5 (-13.8 to 28.8)					
Total PA						0.389	0.326	0.315	0.107	0.275
CG	255.6 (± 11.09)	258.6 (± 9.95)	267.0 (± 11.77)	3.1 (-17.1 to 23.2)	11.5 (-10.8 to 33.7)					
IG	260.5 (± 10.91)	240.5 (± 9.50)	250.0 (± 7.97)	-20.0 (-45.5 to 5.5)	-10.5 (-31.3 to 10.4)					
Diff-in-Diff	---	---	---	-23.1 (-9.4 to 55.6)	-22.0 (-8.5 to 52.5)					

Simple Physical Activity Questionnaire (SIMPAQ) (minutes/week): *n* = 78; CG: *n* = 39; IG: *n* = 39										
Walking						0.839	**≤ 0.001**	0.220	0.755	0.118
CG	35.5 (± 7.64)*¨	64.4 (± 10.3)*	81.8 (± 12.87)¨	28.9 (9.4 to 48.4)	46.4 (22.4 to 70.3)					
IG	51.1 (± 11.78)	69.4 (± 12.94)	68.0 (± 8.21)	18.3 (-10.5 to 47.0)	16.9 (-6.2 to 40.0)					
Diff-in-Diff	---	---	---	-10.6 (-24.1 to 45.4)	-29.5 (-3.9 to 62.7)					
Physical exercises						**0.002**	**0.047**	0.664	**0.025**	0.316
CG	16.7 (± 4.11)¨	17.3 (± 3.01)	22.0 (± 3.33)¨	0.6 (-8.2 to 9.5)	5.3 (-4.6 to 15.3)					
IG	27.9 (± 4.99)	31.1 (± 4.71)	39.7 (± 5.33)	3.2 (-5.5 to 11.8)	11.8 (1.9 to 21.7)					
Diff-in-Diff	---	---	---	2.6 (-14.9 to 9.9)	6.5 (-20.5 to 7.6)					
Other activities						0.873	**0.002**	0.489	**0.051**	0.957
CG	168.1 (± 18.78)*	218.0 (± 16.92)*	186.8 (± 153.69)	49.9 (-1.6 to 101.5)	18.8 (-22.2 to 59.8)					
IG	145.1 (± 17.80)	210.5 (± 21.28)	207.3 (± 25.72)	65.4 (20.7 to 110.1)	62.2 (4.0 to 120.5)					
Diff-in-Diff	---	---	---	15.5 (-83.7 to 52.8)	43.4 (-114.7 to 27.8)					

Vigitel Questionnaire (minutes/week): *n* = 78; CG: *n* = 39; IG: *n* = 39										
Leisure						**≤ 0.001**	0.264	0.598	**0.009**	0.354
CG	96.2 (± 19.27)	119.2 (± 19.63)	126.8 (± 19.18)	23.1 (-13.0 to 59.1)	30.7 (-21.1 to 82.4)					
IG	210.8 (± 84.49)	253.3 (± 26.92)	300.2 (± 33.14)	42.5 (-139.1 to 224.0)	89.5 (-87.3 to 266.3)					
Diff-in-Diff	---	---	---	19.4 (-204.5 to 165.7)	58.8 (-243.0 to 125.4)					
Commuting						**0.046**	0.468	0.961	0.389	0.989
CG	61.5 (± 13.83)	86.3 (± 15.42)	85.9 (± 13.79)	24.8 (-10.0 to 59.5)	24.4 (-13.2 to 62.0)					
IG	120.0 (± 36.52)	136.7 (± 29.46)	134.3 (± 19.68)	16.7 (-42.4 to 75.8)	14.3 (-47.4 to 75.9)					
Diff-in-Diff	---	---	---	-8.1 (-60.4 to 76.7)	-10.1 (-62.1 to 82.3)					
Household						0.091	0.131	0.965	0.563	0.550
CG	208.1 (± 54.09)	214.4 (± 33.39)	263.8 (± 34.75)	6.3 (-77.5 to 90.0)	55.7 (-44.9 to 156.3)					
IG	298.2 (± 54.29)	291.6 (± 48.38)	334.2 (± 34.01)	-6.6 (-143.8 to 130.7)	36.0 (-79.4 to 151.4)					
Diff-in-Diff	---	---	---	-12.9 (-147.9 to 173.6)	-19.7 (-133.3 to 172.8)					
Work						0.345	0.055	0.659	**0.068**	0.324
CG	93.9 (± 63.31)	131.7 (± 40.38)	190.1 (± 61.47)	37.9 (-96.7 to 172.4)	96.3 (-59.9 to 252.4)					
IG	170.8 (± 103.40)	156.5 (± 46.34)	277.7 (± 62.83)	-14.2 (-204.2 to 175.8)	106.9 (-109.3 to 323.1)					
Diff-in-Diff	---	---	---	-52.1 (-180.7 to 284.9)	10.6 (-277.3 to 256.0)					
Total PA						**0.003**	**0.005**	0.750	**0.015**	0.840
CG	459.6 (± 96.18)¨	551.6 (± 57.00)#	666.6 (± 73.70)¨#	92.0 (-99.7 to 283.7)	207.0 (4.2 to 409.8)					
IG	799.7 (± 127.41)	838.1 (± 121.76)	1046.4 (± 115.35)	38.3 (-250.1 to 326.8)	246.6 (-21.8 to 515.1)					
Diff-in-Diff	---	---	---	-53.7 (-292.6 to 399.9)	39.6 (-376.0 to 296.8)					

Bold values indicate statistically significant results. The significance level was set at *p* ≤ 0.05. Simple main effects were examined for significant three-way interaction terms with *p* < 0.10

*PA*PA Physical activity, *MVPA* Moderate-to-vigorous physical activity, *CG* Control group, *IG* Intervention group

x̅ = mean; ± se = standard error; Δ# = post-intervention mean − baseline mean; Δ = difference between post-intervention and baseline; & = difference between intervention group and control group

**Table 6:** Effects of an intervention on physical activity indicators according to sex (intention-to-treat analysis)

	Men					Women				
Indicators	Baseline	Post-intervention	Follow-up	Δ (95% CI)		Baseline	Post-intervention	Follow-up	Δ (95% CI)	
	x̅ (± se)	x̅ (± se)	x̅ (± se)	Δ#	Δ$	x̅ (± se)	x̅ (± se)	x̅ (± se)	Δ#	Δ$
Accelerometry data (minutes/day): *n* = 78; CG: *n* = 39; IG: *n* = 39										
Light PA										
CG	152.2 (± 14.31)	171.5 (± 14.57)	172.7 (± 13.77)	19.3 (-23.1 to 61.8)	20.5 (-20.7 to 61.7)	162.1 (± 6.09)	157.9 (± 4.90)	162.4 (± 4.90)	-4.2 (-19.8 to 11.4)	0.3 (-15.3 to 15.9)
IG	157.7 (± 14.19)*	123.0 (± 13.85)*	143.5 (± 14.99)	-34.7 (-75.9 to 6.5)	-14.2 (-57.1 to 28.7)	163.4 (± 6.96)	159.3 (± 5.87)	155.7 (± 5.05)	-4.2 (-22.3 to 14.0)	-7.7 (-24.9 to 9.4)
Diff-in-Diff	---	---	---	-54.0 (-113.2 to 5.2)	-34.7 (-94.1 to 24.8)	---	---	---	0.0 (-23.9 to 23.9)	-8.0 (-31.2 to 15.1)

Simple Physical Activity Questionnaire (SIMPAQ) (minutes/week): *n* = 78; CG: *n* = 39; IG: *n* = 39										
Physical exercises										
CG	8.3 (± 5.21)	9.5 (± 4.51)	28.7 (± 10.41)	1.2 (-13.1 to 15.5)	20.4 (-3.7 to 44.5)	19.2 (± 5.02)	19.7 (± 3.57)%	19.7(± 2.53)&	0.5 (-11.8 to 12.8)	0.5 (-10.7 to 11.7)
IG	40.6 (± 10.78)	38.0 (± 10.80)	33.0 (± 9.59)	-2.6 (-34.4 to 29.1)	-7.6 (-37.6 to 22.4)	24.1 (± 5.43)¨	29.0 (± 5.13)	41.7 (± 6.26)¨&%	4.9 (-10.0 to 19.8)	17.6 (1.1 to 34.1)
Diff-in-Diff	---	---	---	-3.8 (-38.7 to 31.0)	-28.0 (-66.4 to 10.5)	---	---	---	4.4 (-14.9 to 23.7)	17.1 (-2.9 to 37.1)
Other activities										
CG	135.0 (± 40.28)	240.7 (± 51.60)	192.7 (± 47.34)	105.7 (-30.4 to 241.8)	57.7 (-71.0 to 186.5)	178.0 (± 20.88)	211.2 (± 27.79)	184.8 (± 15.83)	33.2 (-36.1 to 102.5)	6.8 (-45.4 to 59.1)
IG	89.9 (± 17.33)	108.1 (± 17.33)	96.4 (± 21.6)	18.2 (-33.5 to 69.9)	6.5 (-51.6 to 64.7)	161.7 (± 21.62)*	241.2 (± 24.54)*	240.6 (± 30.28)	79.5 (14.3 to 144.7)	78.9 (4.8 to 153.1)
Diff-in-Diff	---	---	---	-87.5 (-233.1 to 58.1)	-51.2 (-192.5 to 90.1)	---	---	---	46.3 (-48.8 to 141.5)	72.1 (-18.6 to 162.8)

Vigitel Questionnaire (minutes/week): *n* = 78; CG: *n* = 39; IG: *n* = 39										
Leisure										
CG	43.3 (± 24.88)%	68.6 (± 30.08)&	161.9 (± 54.14)	25.3 (-55.9 to 106.5)	118.6 (-4.7 to 241.8)	112.0 (± 23.14)%@	134.4 (± 23.16)!	114.7 (± 17.22)+&	22.4 (-42.9 to 87.7)	2.7 (-54.8 to 60.2)
IG	203.3 (± 60.33)	294.7 (± 49.44)%&	226.4 (± 68.11)	91.4 (-70.8 to 253.5)	23.1 (-166.0 to 212.3)	213.0 (± 109.65)	240.8 (± 31.34)%+	322.4 (± 36.98)@!&	27.8 (-199.5 to 255.2)	109.4 (-121.3 to 340.1)
Diff-in-Diff	---	---	---	66.1 (-115.3 to 247.4)	-95.5 (-321.2 to 130.3)	---	---	---	5.4 (-231.1 to 241.9)	106.7 (-131.1 to 344.4)
Work										
CG	100.0 (± 75.43)	225.7 (± 119.13)	398.1 (± 214.16)	125.7 (-167.4 to 418.9)	298.1 (-171.3 to 767.5)	92.0 (± 79.13)	103.5 (± 36.93)	118.4 (± 26.24)&	11.5 (-162.6 to 185.6)	26.4 (-139.8 to 192.6)
IG	0	131.2 (± 68.07)	90.5 (± 43.09)	131.2 (-10.4 to 272.7)	90.5 (0.9 to 180.0)	222.0 (± 133.0)	164.2 (± 56.60)	333.9 (± 77.77)&	-57.8 (-346.0 to 230.3)	111.9 (-195.3 to 419.0)
Diff-in-Diff	---	---	---	5.5 (-320.1 to 330.9)	-207.6 (-685.5 to 270.3)	---	---	---	-69.3 (-406.0 to 267.3)	85.5 (-263.8 to 434.7)
Total PA										
CG	329.4 (±92.5)$	491.8 (± 141.14)	835.0 (± 205.79)$	162.4 (-188.5 to 513.2)	505.5 (38.8 to 972.2)	498.7 (± 121.00)%	569.5 (± 60.44)!	608.6 (± 65.83)&	70.9 (-198.8 to 340.5)	109.9 (-164.7 to 384.6)
IG	508.9 (± 135.59)	687.2 (± 173.01)	651.3 (± 142.82)	178.4 (-278.6 to 635.3)	142.4 (-267.0 to 551.8)	887.0 (± 157.09)	883.3 (± 148.54)	1164.9 (± 136.47)%!&	-3.7 (-434.6 to 427.3)	277.9 (-136.9 to 692.7)
Diff-in-Diff	---	---	---	16.0 (-560.1 to 592.1)	-363.1 (-983.9 to 257.7)	---	---	---	-74.6 (-582.9 to 433.9)	168.0 (-329.5 to 665.5)

*PA* Physical activity, *MVPA* Moderate-to-vigorous physical activity, *CG* Control group, *IG* Intervention group

x̅ = mean; ± se = standard error; Δ# = post-intervention mean − baseline mean; Δ$ = follow-up mean − baseline mean

## Discussion

The aim of this study was to assess the short- (post-intervention) and long-term (follow-up) effects of a PA intervention on various indicators of this behavior and to explore whether sex and age moderate these results in adults with moderate to severe depressive symptoms. While per-protocol analysis indicated possible improvements of the intervention for leisure-time PA, no significant group-by-time interactions were identified in the intention-to-treat analyses. These findings may indicate that adherence plays an important role in intervention engagement and outcomes among individuals experiencing mental distress, although the per-protocol findings should be interpreted cautiously given the potential for selection bias. Regarding the exploratory moderation analyses, intervention effects on leisure-time PA appeared more pronounced among women than men. Similarly, possible intervention effects on walking and physical exercise were observed among older participants, but not among younger counterparts.

There is an increasing demand for more pragmatic randomized controlled trials [41]. Such studies provide high external validity, enabling the generalization of results to real-world clinical practice due to their methodological alignment with the norms of daily life [41]. In the *Vincular* Project, for instance, the meetings were designed to align with the participants’ profiles, considering, whenever possible, their personal preferences and prior experiences. Although the activity schedule was planned prior to the intervention, flexibility was built into the protocol, allowing participants to adjust the intensity of activities based on their physical and psychological conditions as well as their perceived capabilities. However, despite these pragmatic characteristics, the intervention also involved substantial staff support, individualized follow-up, and implementation within an academic setting, which should be considered when interpreting its external validity and potential applicability to other contexts.

The *Vincular* Project was effective on improving leisure-time PA - but not commuting and labor-related PA, as well as accelerometer assessed PA - among adults with moderate to severe depressive symptoms. Even though the effect of PA on reducing depressive symptoms is well established [4–6, 8], the literature remains inconsistent regarding the outcomes of clinical trials aimed at increasing PA levels in individuals with depression [17, 18]. According to a systematic review, of 16 controlled clinical trials, only seven (47%) reported significant improvements in PA, with just one of them using accelerometry as a measurement tool [17]. One possible interpretation is that participants became more aware of different domains of PA throughout the intervention, which may have influenced how they reported these behaviors, particularly in self-reported measures. This process of awareness may have been fostered by the reflective strategies adopted in the intervention, based on Self-Determination Theory [42], which aimed to promote participants’ autonomy, competence, and sense of belonging in relation to health behaviors. It is known that factors such as enjoyment, skill mastery, autonomous motivation, social interaction, and group bonding are important mediators in the relationship between PA and mental health [20, 26], and these are more commonly present in the leisure and transportation domains than in domestic and occupational settings [28]. By reflecting on the role of PA in their daily routines, participants may have become more aware of which PA practices were more compatible with their individual contexts and preferences. Moreover, because the leisure domain typically involves greater autonomy and enjoyment, it may have generated higher motivation and engagement, encouraging the incorporation of new activities and amplifying the effects of the intervention within this specific context. However, because accelerometer-based outcomes did not demonstrate comparable effects, these interpretations remain speculative and should be viewed cautiously.

Although the moderating effects of sex and age on PA outcomes were explored, this relationship does not appear to be fully understood and may vary depending on the context, particularly in individuals with depressive symptoms. Schuch and Stubbs [7] highlight the importance of identifying which patients are most likely to benefit from the antidepressant effects of exercise, considering potential moderators of this relationship. In this regard, the impact of PA on depression may differ between men and women and vary according to biological and psychosocial factors that influence the response to exercise [43]. Exploratory analyses suggested that the effects may be more pronounced among women and older adults; however, the mechanisms underlying these differences were not directly assessed in this study. Although features such as social interaction and the autonomy-supportive nature of the intervention may have contributed to engagement in these subgroups, these interpretations remain speculative and should be confirmed in future studies specifically designed to investigate potential moderators and mechanisms.

When addressing individuals with clinical conditions such as depressive symptoms and their intervention possibilities, a key question arises: what approach is more effective – initially focusing on improving disease-related indicators before increasing PA levels, or the reverse? This question is particularly relevant given the bidirectional relationship between PA and depression [44]. Despite the well-documented benefits of a healthy lifestyle, individuals with depressive symptoms face significant barriers, including the symptoms of the condition itself, which often lead to a lack of motivation and loss of interest [45]. These barriers are common and frequently result in participants dropping out of health promotion programs across various contexts, a phenomenon also observed in the present study, where a high dropout rate was reported among intervention participants. Adopting and maintaining healthy behaviors is inherently challenging for clinical populations, and individuals with depressive symptoms are no exception [7]. A meta-analysis and meta-regression by Stubbs et al. [46] reported an average dropout rate of 18.1% in randomized clinical trials involving exercise for individuals with depression, a value considerably lower than the rate observed in the *Vincular* Project. To minimize dropouts, several strategies were implemented, including designing the intervention around Self-Determination Theory, which emphasizes addressing basic psychological needs to enhance intrinsic motivation. Additionally, the supervised intervention model and direct, personalized contact between professionals and participants – particularly those with higher rates of absence – were integral components of the approach. Even so, future trials should prioritize greater adaptation to individual needs from the outset, taking into account barriers to PA and other contributing factors. This individualized approach could help mitigate dropout rates and enhance adherence to health promotion interventions.

It is essential to recognize the limitations of this study and address them in future interventions. While the *Vincular* Project aimed to modify active behaviors, the primary outcome focused on depressive symptoms, with PA as a secondary outcome, alongside other components such as sedentary behavior and sleep. Additionally, the multiple PA outcomes and exploratory subgroup analyses increase the risk of type I error, despite the use of Bonferroni post hoc adjustments. Therefore, the findings should be interpreted with caution. Although detailed methodological information regarding the intervention sessions is provided in preprint and supplementary materials, and the study protocol was followed without deviations, the reproducibility of the project may still be limited. This is due to the personalized nature of the sessions and the flexibility in how professionals approached and delivered the proposed content, which could influence participants’ engagement and outcomes. Additionally, the study benefited from academic partnerships and an infrastructure that may not be easily replicated outside a research context, thus limiting the generalizability of the findings in other contexts and populations.

A further limitation concerns the high attrition observed throughout the study, particularly in the intervention group, which presented a dropout rate of 59%. This may reflect both the intensity of the intervention (two 90-minute sessions per week over 16 weeks) and intrinsic barriers faced by individuals with depressive symptoms, such as anhedonia, lack of motivation, and low energy. Such attrition may have influenced the feasibility of the intervention and the interpretation of the findings, particularly given the differences observed between per-protocol and intention-to-treat analyses. Moreover, comparisons between participants who completed the intervention and those who did not (Additional file 4) indicated that completers had lower depressive symptom severity at baseline, as reflected by PHQ-9 scores. This suggests that individuals with higher PHQ-9 scores at baseline may have been less likely to adhere to the intervention, which should be considered when interpreting the results. Consequently, the per-protocol findings should be interpreted cautiously, as they may partially reflect characteristics of participants who were able to remain engaged in the intervention, introducing the possibility of selection bias.

Future clinical trials aiming to improve mental health outcomes through active behaviors should consider baseline behavioral patterns when performing group allocation. In the present study, although groups were generally comparable at baseline, total PA assessed by questionnaire was higher in the intervention group. This baseline difference should be considered when interpreting findings related to self-reported PA, as it may have influenced the magnitude of observed changes. However, intervention effects were estimated using repeated-measures GEE models, which incorporate baseline values and focus on changes over time between groups. Another limitation relates to the divergent results observed depending on the method used to assess PA. In our study, substantial differences between the intervention and control groups were found when PA was measured using self-reported questionnaires, but not when assessed by accelerometers. This discrepancy may be partly explained by the timing of the accelerometer assessment, which captured behavior during the week following the end of the intervention, rather than during the active intervention period. Accordingly, these data may be better interpreted as reflecting short-term maintenance of behavior rather than immediate intervention effects. It is also plausible that participants in the intervention group adopted less active behavior during this period as a form of rest, following an intense sequence of structured activities. Conversely, the self-reported questions referred to PA performed before the second data collection, which may better reflect the participants’ behaviors during the intervention itself.

Despite this, this study presented relevant contributions to clarify the literature and the reality on promoting healthier life-style changes among individuals under distress. The *Vincular* Project represented a relatively novel unconventional PA interventions for adults with moderate to severe depressive symptoms, as the structure of the program differed from traditional exercise-based clinical trials that typically focus solely on aerobic, strength, or combined training protocols. Instead, the *Vincular* Project offered diverse sessions throughout the program, allowing participants to explore various movement practices and potentially increasing opportunities for engagement and personal relevance. It is also worth highlighting the strong design of the study: a theory-guided, assessor-blinded, randomized controlled trial. Self-Determination Theory provides a solid framework for the development of behavioral interventions, offering structured support for autonomy, competence, and relatedness: the basic psychological needs that facilitate the adoption and maintenance of behavioral changes [47]. These needs are inherently connected to intrapersonal (e.g., motivation, self-efficacy), interpersonal (e.g., relationships, social support), and environmental (e.g., access to resources, contextual flexibility) factors that influence the adoption of a healthier lifestyle. Despite this integrative potential, the application of Self-Determination Theory in clinical trials remains limited, particularly in studies targeting mental health outcomes. This theoretical model is especially valuable in the non-pharmacological treatment of depression, as individuals with depressive symptoms often experience frustration of their psychological needs, which contributes to demotivation, poor engagement with treatment, and symptom persistence. Finally, this study investigated both short- and long-term effects and their potential moderators, and employed a comprehensive assessment of PA, including both total and contextual PA, measured through self-report and accelerometry.

## Conclusions

The per-protocol analysis suggested positive effects of the intervention on leisure-time PA, with increases observed during the intervention period. However, these effects were not confirmed in the intention-to-treat analysis. Exploratory moderator analyses indicated potential differences according to sex and age, but these findings should be interpreted with caution and considered hypothesis-generating. Overall, the results highlight the challenges of adherence and the importance of considering individual variability when designing PA interventions for adults with depressive symptoms. Future programs should prioritize strategies to improve adherence, given the high likelihood of dropout among individuals experiencing psychological distress.

## Supplementary Information

Supplementary Material 1.

Supplementary Material 2.

Supplementary Material 3.

Supplementary Material 4.

## Data Availability

The data that support the findings of this study are available from the corresponding author, CB, upon reasonable request.

## Abbreviations

ANOVA: Analysis of variance
BDNF: Brain-derived neurotrophic factor
CONSORT: Consolidated Standards of Reporting Trials
ENMO: Euclidean Norm Minus One
GEE: Generalized Estimating Equations
PA: Physical activity
PHQ-9: Patient Health Questionnaire
ReBEC: Brazilian Clinical Trials Registry
SIMPAQ: Simple Physical Activity Questionnaire
SPSS: Statistical Package for the Social Sciences
TIDieR: Template for Intervention Description and Replication
TNF-α: Tumor necrosis factor-alpha
VIGITEL: Surveillance of Risk and Protective Factors for Chronic Diseases by Telephone Survey

## References

[1] Cao L, Li X, Yan P, Wang X, Li M, Li R, et al. The effectiveness of aerobic exercise for hypertensive population: a systematic review and meta-analysis. J Clin Hypertens. 2019;21(7):868–76. 10.1111/jch.13583. PMC8030461 31169988

[2] Leonel LS, Brum G, Alberton CL, Delevatti RS. Aquatic training improves HbA1c, blood pressure and functional outcomes of patients with type 2 diabetes: a systematic review with meta-analysis. Diabetes Res Clin Pract. 2023;197:110575. 10.1016/j.diabres.2023.110575. 36780954

[3] Mustian KM, Alfano CM, Heckler C, Kleckner AS, Kleckner IR, Leach CR, et al. Comparison of pharmaceutical, psychological, and exercise treatments for cancer-related fatigue. JAMA Oncol. 2017;3(7):961. 10.1001/jamaoncol.2016.6914. PMC5557289 28253393

[4] Heissel A, Heinen D, Brokmeier LL, Skarabis N, Kangas M, Vancampfort D, et al. Exercise as medicine for depressive symptoms? A systematic review and meta-analysis with meta-regression. Br J Sports Med. 2023;57(16):1049–57. 10.1136/bjsports-2022-106282. PMC10423472 36731907

[5] Pearce M, Garcia L, Abbas A, Strain T, Schuch FB, Golubic R, et al. Association between physical activity and risk of depression. JAMA Psychiatry. 2022;79(6):550. 10.1001/jamapsychiatry.2022.0609. PMC9008579 35416941

[6] Recchia F, Leung CK, Chin EC, et al. Comparative effectiveness of exercise, antidepressants and their combination in treating non-severe depression: a systematic review and network meta-analysis of randomised controlled trials. Br J Sports Med. 2022;56(23):1375–80. 10.1136/bjsports-2022-105964. PMC9685718 36113975

[7] Schuch FB, Stubbs B. The role of exercise in preventing and treating depression. Curr Sports Med Rep. 2019;18(8):299–304. 10.1249/jsr.0000000000000620. 31389872

[8] Singh B, Olds T, Curtis R, et al. Effectiveness of physical activity interventions for improving depression, anxiety and distress: an overview of systematic reviews. Br J Sports Med. 2023;57(18):1203–9. 10.1136/bjsports-2022-106195. PMC10579187 36796860

[9] Carneiro LF, Mota MP, Schuch F, Deslandes A, Vasconcelos-Raposo J. Portuguese and Brazilian guidelines for the treatment of depression: exercise as medicine. Braz J Psychiatry. 2017;40(2):210–1. 10.1590/1516-4446-2017-2272. PMC6900767 28876380

[10] Ross RE, Van Derwerker CJ, Saladin ME, Gregory CM. The role of exercise in the treatment of depression: biological underpinnings and clinical outcomes. Mol Psychiatry. 2022;28(1):298–328. 10.1038/s41380-022-01819-w. PMC9969795 36253441

[11] Schuch FB, Morres ID, Ekkekakis P, Rosenbaum S, Stubbs B. A critical review of exercise as a treatment for clinically depressed adults: time to get pragmatic. Acta Neuropsychiatr. 2017;29(2):65–71. 10.1017/neu.2016.21. 27145824

[12] Schuch FB, Vancampfort D, Richards J, Rosenbaum S, Ward PB, Stubbs B. Exercise as a treatment for depression: a meta-analysis adjusting for publication bias. J Psychiatr Res. 2016;77:42–51. 10.1016/j.jpsychires.2016.02.023. 26978184

[13] Schuch FB, Vancampfort D, Firth J, et al. Physical activity and incident depression: a meta-analysis of prospective cohort studies. Am J Psychiatry. 2018;175(7):631–48. 10.1176/appi.ajp.2018.17111194. 29690792

[14] Schuch FB, Vancampfort D, Firth J, et al. Physical activity and sedentary behavior in people with major depressive disorder: a systematic review and meta-analysis. J Affect Disord. 2017;210:139–50. 10.1016/j.jad.2016.10.050. 28033521

[15] Stubbs B, Koyanagi A, Schuch FB, et al. Physical activity and depression: a large cross-sectional, population-based study across 36 low- and middle-income countries. Acta Psychiatr Scand. 2016;134(6):546–56. 10.1111/acps.12654. 27704532

[16] Vancampfort D, Firth J, Schuch FB, Rosenbaum S, Mugisha J, Hallgren M, et al. Sedentary behavior and physical activity levels in people with schizophrenia, bipolar disorder and major depressive disorder: a global systematic review and meta-analysis. World Psychiatry. 2017;16(3):308–15. 10.1002/wps.20458. PMC5608847 28941119

[17] Ashdown-Franks G, Williams J, Vancampfort D, et al. Is it possible for people with severe mental illness to sit less and move more? A systematic review of interventions to increase physical activity or reduce sedentary behaviour. Schizophr Res. 2018;202:3–16. 10.1016/j.schres.2018.06.058. 29980329

[18] Stanton R, Reaburn P. Exercise and the treatment of depression: a review of the exercise program variables. J Sci Med Sport. 2014;17(2):177–82. 10.1016/j.jsams.2013.03.010. 23602562

[19] McKenna C, Moyo B, Goodwin J. Barriers to using physical exercise as an intervention within inpatient mental health settings: a systematic review. Int J Ment Health Nurs. 2024;33(4):817–33. 10.1111/inm.13302. 38332557

[20] Teychenne M, White RL, Richards J, Schuch FB, Rosenbaum S, Bennie JA. Do we need physical activity guidelines for mental health: What does the evidence tell us? Ment Health Phys Act. 2020;18:100315. 10.1016/j.mhpa.2019.100315.

[21] Mammen G, Faulkner G. Physical activity and the prevention of depression: a systematic review of prospective studies. Am J Prev Med. 2013;45(5):649–57. 10.1016/j.amepre.2013.08.001. 24139780

[22] Morgan F, Battersby A, Weightman AL, et al. Adherence to exercise referral schemes by participants–what do providers and commissioners need to know? A systematic review of barriers and facilitators. BMC Public Health. 2016;16:1–11. 10.1186/s12889-016-2882-7. PMC4779205 26944952

[23] Ravindran AV, Balneaves LG, Faulkner G, et al. Clinical guidelines for the management of adults with major depressive disorder: Sect. 5. Complementary and alternative medicine treatments. Can J Psychiatry. 2016;61(9):576–87. 10.1177/0706743716660290. PMC4994794 27486153

[24] Gordon BR, McDowell CP, Hallgren M, Meyer JD, Lyons M, Herring MP. Association of efficacy of resistance exercise training with depressive symptoms: meta-analysis and meta-regression analysis of randomized clinical trials. JAMA Psychiatry. 2018;75(6):566–76. 10.1001/jamapsychiatry.2018.0572. PMC6137526 29800984

[25] Matias TS, Lopes MVV, da Costa BGG, Silva KS, Schuch FB. Relationship between types of physical activity and depression among 88,522 adults. J Affect Disord. 2022;297:415–20. 10.1016/j.jad.2021.10.051. 34715174

[26] White RL, Parker PD, Lubans DR, MacMillan F, Olson R, Astell-Burt T, et al. Domain-specific physical activity and affective wellbeing among adolescents: an observational study of the moderating roles of autonomous and controlled motivation. Int J Behav Nutr Phys Act. 2018;15:1–13. 10.1186/s12966-018-0722-0. PMC6131748 30200980

[27] Lopes MVV, Matias TS, da Costa BGG, Schuch FB, Chaput JP, Silva KS. The relationship between physical activity and depressive symptoms is domain-specific, age-dependent, and non-linear: an analysis of the Brazilian national health survey. J Psychiatr Res. 2023;159:205–12. 10.1016/j.jpsychires.2023.01.041 36739848

[28] Teychenne M, Abbott G, Lamb KE, Rosenbaum S, Ball K. Is the link between movement and mental health a two-way street? Prospective associations between physical activity, sedentary behaviour and depressive symptoms among women living in socioeconomically disadvantaged neighbourhoods. Prev Med. 2017;102:72–8. 10.1016/j.ypmed.2017.07.005. 28694061

[29] Schuch FB, Dunn AL, Kanitz AC, Delevatti RS, Fleck MP. Moderators of response in exercise treatment for depression: a systematic review. J Affect Disord. 2016;195:40–9. 10.1016/j.jad.2016.01.014. 26854964

[30] Hopewell S, Chan A, Gary SC, et al. CONSORT 2025 statement: updated guideline for reporting randomised trials. BMJ. 2025;389:e081123. 10.1136/bmj-2024-081123. PMC11995449 40228833

[31] Bertuol C, Alves DK, Del Duca GF. Vincular Project: study protocol for a randomized controlled trial with changes in 24-h movement behaviors targeting the reduce of depressive symptoms in adults. Trials. 27(1). 10.1186/s13063-026-09771-8. PMC13343858 42141487

[32] Santos IS, Tavares BF, Munhoz TN, et al. Sensibilidade e especificidade do Patient Health Questionnaire-9 (PHQ-9) entre adultos da população geral. Cad Saude Publica. 2013;29:1533–43. 10.1590/0102-311x00144612. 24005919

[33] Deci EL, Ryan RM. The what and why of goal pursuits: human needs and the self-determination of behavior. Psychol Inq. 2000;11(4):227–68. 10.1207/s15327965pli1104_01.

[34] Ryan RM, Deci EL. Self-determination theory and the facilitation of intrinsic motivation, social development, and well-being. Am Psychol. 2000;55(1):68. 10.1037/0003-066x.55.1.68. 11392867

[35] Bertuol C, Schuch FB, Tozetto WR, Alves DK, Del Duca GF. Effectiveness of a physical activity intervention based on self-determination theory on depressive symptoms in Brazilian adults with heightened depressive symptoms: a randomised clinical trial. Int J Sport Exerc Psychol. 2025;1–20. 10.1080/1612197x.2025.2590493.

[36] Monteiro CA, Moura EC, Jaime PC, et al. Monitoramento de fatores de risco para doenças crônicas por entrevistas telefônicas. Rev Saude Publica. 2005;39(1):47–57. 10.1590/s0034-89102005000100007. 15654460

[37] Rosenbaum S, Morell R, Abdel-Baki A, et al. Assessing physical activity in people with mental illness: 23-country reliability and validity of the simple physical activity questionnaire (SIMPAQ). BMC Psychiatry. 2020;20:1–12. 10.1186/s12888-020-2473-0. PMC7060599 32143714

[38] van Hees VT, Gorzelniak L, Dean León EC, et al. Separating movement and gravity components in an acceleration signal and implications for the assessment of human daily physical activity. PLoS ONE. 2013;8(4):e61691. 10.1371/journal.pone.0061691. PMC3634007 23626718

[39] Hildebrand MVHV, van Hees VT, Hansen BH, Ekelund ULF. Age group comparability of raw accelerometer output from wrist- and hip-worn monitors. Med Sci Sports Exerc. 2014;46(9):1816–24. 10.1249/MSS.0000000000000289. 24887173

[40] van Hees VT, Sabia S, Jones SE, et al. Estimating sleep parameters using an accelerometer without sleep diary. Sci Rep. 2018;8(1):1–11. 10.1038/s41598-018-31266-z. PMC6113241 30154500

[41] Schuch FB, Morres ID, Ekkekakis P, Rosenbaum S, Stubbs B. Exercise works for depression: bridging the implementation gap and making exercise a core component of treatment. Acta Neuropsychiatr. 2017;29(2):124–6. 10.1017/neu.2017.1. 28262083

[42] Teixeira PJ, Carraça EV, Markland D, Silva MN, Ryan RM. Exercise, physical activity, and self-determination theory: a systematic review. Int J Behav Nutr Phys Act. 2012;9(1):78. 10.1186/1479-5868-9-78. PMC3441783 22726453

[43] Kandola A, Ashdown-Franks G, Hendrikse J, Sabiston CM, Stubbs B. Physical activity and depression: towards understanding the antidepressant mechanisms of physical activity. Neurosci Biobehav Rev. 2019;107:525–39. 10.1016/j.neubiorev.2019.09.040. 31586447

[44] Hiles SA, Lamers F, Milaneschi Y, Penninx BWJH. Sit, step, sweat: longitudinal associations between physical activity patterns, anxiety and depression. Psychol Med. 2017;47(8):1466–77. 10.1017/s0033291716003548. 28137333

[45] World Health Organization. Mental health atlas 2017. Geneva: World Health Organization; 2018.

[46] Stubbs B, Vancampfort D, Rosenbaum S, et al. Dropout from exercise randomized controlled trials among people with depression: a meta-analysis and meta regression. J Affect Disord. 2016;190:457–66. 10.1016/j.jad.2015.10.019. 26551405

[47] Gillison FB, Rouse P, Standage M, Sebire SJ, Ryan RM. A meta-analysis of techniques to promote motivation for health behaviour change from a self-determination theory perspective. Health Psychol Rev. 2019;13(1):110–30. 10.1080/17437199.2018.1534071. 30295176

