# Supplementary material for: Effects and moderators of a 16-week intervention on contextual physical activity in adults with moderate to severe depressive symptoms: short and long-term results of a randomized clinical trial
Source: Int J Behav Nutr Phys Act. 2026 Jul 21;23:97. doi: 10.1186/s12966-026-01950-9 (PMC13679006; doi:10.1186/s12966-026-01950-9)
Supplement: Supplementary file 3 [file 12966_2026_1950_MOESM3_ESM.docx]

**Table 1.** Effects and moderators of the intervention on leisure-time PA among insufficiently active participants at baseline.

| **Indicators** | **Baseline** | **Post-intervention** | **Follow-up** | **Δ (95% CI)** | |  | **p-value** | | | | |
| --- | --- | --- | --- | --- | --- | --- | --- | --- | --- | --- | --- |
|  | **x̅ (±se)** | **x̅ (±se)** | **x̅ (±se)** | **Δ#** | **Δ$** |  | **g** | **t** | **g*t** | **g*t*sex** | **g*t*age** |
|  | **Per-protocol analysis (n = 24; CG: n = 14; IG: n = 10)** | | | | | | | | | | |
| **Leisure** |  |  |  |  |  |  | 0.216 | **<0.001** | **<0.001** | 0.169 | 0.822 |
| CG | 37.5 (±14.27) | 65.7 (±20.65)& | 100.7 (±43.14) | 28.2 (-17.4 to 73.8) | 63.2 (-26.3 to 152.7) |  |  |  |  |  |  |
| IG | 12.0 (±11.38)% | 167.5 (±20.92)&% | 110.0 (±45.30) | 155.5 (113.2 to 197.8) | 98.0 (2.5 to 193.5) |  |  |  |  |  |  |
| Diff-in-Diff | --- | --- | --- | 127.3 (-189.5 to -65.1) | 34.8 (-165.7 to 96.1) |  |  |  |  |  |  |
|  | **Intention-to-treat analysis (n = 52; CG: n = 27; IG: n = 25)** | | | | | | | | | | |
| **Leisure** |  |  |  |  |  |  | **<0.001** | **<0.001** | **<0.001** | **0.015** | 0.407 |
| CG | 23.1 (±8.70)*¨ | 66.3 (±14.88)&* | 117.4 (±24.20)$¨ | 43.2 (10.8 to 75.6) | 94.2 (42.8 to 145.7) |  |  |  |  |  |  |
| IG | 30.6 (±9.99)%# | 211.2 (±26.68)&% | 246.4 (±38.34)$# | 180.6 (127.6 to 233.7) | 215.8 (140.0 to 291.6) |  |  |  |  |  |  |
| Diff-in-Diff | --- | --- | --- | 137.4 (-199.6 to -75.2) | 121.6 (-213.1 to -29.9) |  |  |  |  |  |  |

Notes: PA = physical activity; CG = control group; IG = intervention group; x̅ = mean; ± se = standard error; Δ# = post-intervention mean − baseline mean; Δ$ = follow-up mean − baseline mean.

**Table 2.** Effects of the intervention on leisure-time PA among insufficiently active participants, according to sex (intention-to-treat analysis).

| **Indicators** | **Men** | | | | |  | **Women** | | | | |
| --- | --- | --- | --- | --- | --- | --- | --- | --- | --- | --- | --- |
|  | **Baseline** | **Post-intervention** | **Follow-up** | **Δ (95% CI)** | |  | **Baseline** | **Post-intervention** | **Follow-up** | **Δ (95% CI)** | |
|  | **x̅ (±se)** | **x̅ (±se)** | **x̅ (±se)** | **Δ#** | **Δ$** |  | **x̅ (±se)** | **x̅ (±se)** | **x̅ (±se)** | **Δ#** | **Δ$** |
|  | **Simple Physical Activity Questionnaire (SIMPAQ) (minutes/week): n = 36; CG: n = 20; IG: n = 16** | | | | | | | | | | |
| **Leisure** |  |  |  |  |  |  |  |  |  |  |  |
| CG | 20.6  (±14.26) | 46.0  (±23.82) | 171.2  (±63.55) | 25.3  (-21.0 to 71.6) | 150.5  (18.3 to 282.7) |  | 24.2  (±10.79)@ | 74.9  (±18.26) | 94.7  (±19.37)@ | 50.7  (9.4 to 92.0) | 70.5  (27.4 to 113.7) |
| IG | 30.0 (±25.98)@ | 206.8  (±75.18)@$ | 110.3  (±71.97)$ | 176.8  (76.1 to 277.6) | 80.3  (-12.4 to 173.1) |  | 30.7  (±10.81)*¨ | 212.1  (±28.35)*$ | 272.3  (±41.18)¨$ | 181.4  (121.2 to 241.6) | 241.6  (157.5 to 325.7) |
| Diff-in-Diff | --- | --- | --- | 151.5  (-262.4 to -40.6) | -70.2  (-91.3 to 231.6) |  | --- | --- | --- | 130.7  (-203.6 to -57.6) | 171.1  (-265.6 to -76.5) |

Notes: PA = physical activity; CG = control group; IG = intervention group; x̅ = mean; ± se = standard error; Δ# = post-intervention mean − baseline mean; Δ$ = follow-up mean − baseline mean.
