# Supplementary material for: Effects and moderators of a 16-week intervention on contextual physical activity in adults with moderate to severe depressive symptoms: short and long-term results of a randomized clinical trial
Source: Int J Behav Nutr Phys Act. 2026 Jul 21;23:97. doi: 10.1186/s12966-026-01950-9 (PMC13679006; doi:10.1186/s12966-026-01950-9)
Supplement: Supplementary file 4 [file 12966_2026_1950_MOESM4_ESM.docx]

**Table 1.** Baseline characteristics of participants according to intervention completion status.

| **Variables** | **Intervention group (n = 39)** | | **p value** |  | **Total sample (n = 78)** | | **p value** |
| --- | --- | --- | --- | --- | --- | --- | --- |
|  | **Completers (n = 16)** | **Non-completers (n = 23)** |  |  | **Completers (n = 47)** | **Non-completers (n = 31)** |  |
|  | **x̅ (± sd)** | **x̅ (± sd)** |  |  | **x̅ (± sd)** | **x̅ (± sd)** |  |
| Age | 36.1 (±10.04) | 38.0 (±10.60) | 0.718 |  | 36.1 (±10.19) | 39.5 (±10.05) | 0.148 |
| Educactional level (years) | 14.9 (±4.60) | 18.9 (±4.13) | 0.493 |  | 15.6 (±2.42) | 16.5 (±6.38) | 0.389 |
| Light PA* | 155.4 (±38.99) | 163.9 (±41.16) | 0.521 |  | 155.1 (±38.16) | 167.0 (±39.10) | 0.185 |
| Moderate-to-vigorous PA* | 103.6 (±77.46) | 89.8 (±36.56) | 0.461 |  | 99.5 (±58.64) | 87.7 (±34.27) | 0.315 |
| Total PA* | 259.0 (±85.33) | 253.7 (±57.48) | 0.818 |  | 254.6 (±78.40) | 254.7 (±55.30) | 0.994 |
| PHQ-9 total score | 14.9 (±4.60) | 18.9 (±4.13) | 0.007 |  | 16.4 (±4.33) | 18.8 (±4.06) | 0.014 |
|  | **n (%)** | **n (%)** |  |  | **n (%)** | **n (%)** |  |
| Sex |  |  | 0.444 |  |  |  | 0.282 |
| Male | 5 (31.3) | 4 (17.4) |  |  | 13 (27.7) | 5 (16.1) |  |
| Female | 11 (68.2) | 19 (82.6) |  |  | 34 (72.3) | 26 (83.9) |  |
| Marital status |  |  | 0.711 |  |  |  | 0.445 |
| With a partner | 5 (31.3) | 5 (21.7) |  |  | 11 (23.4) | 10 (32.3) |  |
| Without a partner | 11 (68.7) | 18 (78.3) |  |  | 36 (76.6) | 21 (67.7) |  |
| Self-reported skin color |  |  | 0.495 |  |  |  | 0.448 |
| White | 12 (75.0) | 14 (60.9) |  |  | 35 (74.5) | 20 (64.5) |  |
| Other+ | 4 (25.0) | 9 (39.1) |  |  | 12 (25.5) | 11 (35.5) |  |
| Diagnosis of mental illness |  |  | 1.000 |  |  |  | 0.810 |
| No | 7 (43.8) | 9 (39.1) |  |  | 16 (34.0) | 12 (38.7) |  |
| Yes | 9 (56.2) | 14 (60.9) |  |  | 31 (66.0) | 19 (61.3) |  |
| Depression diagnosis |  |  | 0.752 |  |  |  | 0.355 |
| No | 8 (50.0) | 13 (56.5) |  |  | 23 (48.9) | 19 (61.3) |  |
| Yes | 8 (50.0) | 10 (43.5) |  |  | 24 (51.1) | 12 (38.7) |  |
| Use of medication for depression |  |  | 0.503 |  |  |  | 1.000 |
| No | 7 (43.8) | 7 (30.4) |  |  | 16 (34.0) | 10 (32.3) |  |
| Yes | 9 (56.2) | 16 (69.6) |  |  | 31 (66.0) | 21 (67.7) |  |
| Participation on psychotherapy |  |  | 0.289 |  |  |  | 0.671 |
| No | 2 (12.5) | 8 (34.8) |  |  | 11 (23.4) | 10 (32.3) |  |
| No, but I've done it before | 6 (37.5) | 6 (26.1) |  |  | 20 (42.6) | 11 (35.4) |  |
| Yes | 8 (50.0) | 9 (39.1) |  |  | 16 (34.0) | 10 (32.3) |  |
| Family history of depression |  |  | 0.867 |  |  |  | 0.609 |
| No | 2 (12.5) | 4 (17.4) |  |  | 6 (12.8) | 4 (12.9) |  |
| Yes | 13 (81.2) | 17 (73.9) |  |  | 38 (80.8) | 23 (74.2) |  |
| Didn't know the answer | 1 (6.3) | 2 (8.7) |  |  | 3 (6.4) | 4 (12.9) |  |

Notes: PA = physical activity; * = Accelerometry data in in/day; + = Black, Brown (Parda), Yellow (Asian descent), Indigenous, Other; x̄ = mean; ±sd = standard deviation; n = absolute frequency; % = relative frequency; p-values were obtained using the t-test and chi-squared test.
